# COVID-19 vaccine development: milestones, lessons and prospects

**DOI:** 10.1038/s41392-022-00996-y

**Published:** 2022-05-03

**Authors:** Maochen Li, Han Wang, Lili Tian, Zehan Pang, Qingkun Yang, Tianqi Huang, Junfen Fan, Lihua Song, Yigang Tong, Huahao Fan

**Affiliations:** 1 College of Life Science and Technology, Beijing University of Chemical Technology, Beijing, China; 2 Laboratory for Clinical Immunology, Harbin Children’s Hospital, Harbin, China; 3 College of Materials Science and Engineering, Beijing University of Chemical Technology, Beijing, China; 4 Institute of Cerebrovascular Disease Research and Department of Neurology, Xuanwu Hospital of Capital Medical University, Beijing, China; 5 Beijing Advanced Innovation Center for Soft Matter Science and Engineering, Beijing University of Chemical Technology, Beijing, China

**Keywords:** Vaccines, Infectious diseases

## Abstract

With the constantly mutating of SARS-CoV-2 and the emergence of Variants of Concern (VOC), the implementation of vaccination is critically important. Existing SARS-CoV-2 vaccines mainly include inactivated, live attenuated, viral vector, protein subunit, RNA, DNA, and virus-like particle (VLP) vaccines. Viral vector vaccines, protein subunit vaccines, and mRNA vaccines may induce additional cellular or humoral immune regulations, including Th cell responses and germinal center responses, and form relevant memory cells, greatly improving their efficiency. However, some viral vector or mRNA vaccines may be associated with complications like thrombocytopenia and myocarditis, raising concerns about the safety of these COVID-19 vaccines. Here, we systemically assess the safety and efficacy of COVID-19 vaccines, including the possible complications and different effects on pregnant women, the elderly, people with immune diseases and acquired immunodeficiency syndrome (AIDS), transplant recipients, and cancer patients. Based on the current analysis, governments and relevant agencies are recommended to continue to advance the vaccine immunization process. Simultaneously, special attention should be paid to the health status of the vaccines, timely treatment of complications, vaccine development, and ensuring the lives and health of patients. In addition, available measures such as mix-and-match vaccination, developing new vaccines like nanoparticle vaccines, and optimizing immune adjuvant to improve vaccine safety and efficacy could be considered.

## Introduction

Severe acute respiratory syndrome coronavirus 2 (SARS-CoV-2) is a highly infectious positive-sense, single-stranded RNA virus that spreads rapidly worldwide. The resulting infection, known as coronavirus disease 2019 (COVID-19), can cause several symptoms, such as cough, fever, chest discomfort, and even respiratory distress syndrome in severe cases.^1,2^ As of March 28, 2022, there were 480,905,839 confirmed cases of COVID-19 worldwide, and 6,123,493 patients died of viral infection or other related complications (https://coronavirus.jhu.edu/).

Effective and safe vaccines are essential to control the COVID-19 pandemic.^3,4^ Several studies have reported the progress in developing SARS-CoV and Middle East respiratory syndrome coronavirus (MERS-CoV) vaccines.^5–8^ The preclinical data of these candidate vaccines partly saved the time for developing the current marketed SARS-CoV-2 vaccines and would provide platforms for the future widespread application of SARS-CoV-2 vaccines. The World Health Organization (WHO) classifies COVID-19 vaccines that have been analyzed or approved for clinical trials into the following categories: inactivated vaccine, live attenuated, vector, RNA, DNA, protein subunit, and virus-like particle (VLP) vaccines.

Animal experiments play a critical role in vaccine development, including evaluating the safety and protective efficacy, determining the injection schedule, and establishing the effective dosage. Small animals, especially rodents, are the foundation of biological and immunological studies in vaccine development.^9,10^ Generally, rats, mice, guinea pigs, rabbits, and other animals can be used as animal models to evaluate candidate vaccines’ immunogenicity, tolerance, and safety. However, due to species differences between these animals and humans, similar biological effects may not be produced after vaccination. The studies of non-human primates (NHPs) are helpful in understanding and illustrating human immune responses, owing to similar innate and adaptive immune responses.^9^ Many reagents used to identify human immune molecules also show similar effects on NHPs. In addition to preclinical trials (animal experiments), clinical trials are essential for developing vaccines. The safety, dosage, and tolerance of vaccines are assessed in the Phase I trial, efficacy and adverse effects are investigated in Phase II and III trials.

Vaccination is a pivotal means to prevent the spread of SARS-CoV-2 and ultimately quell the pandemic. However, vaccine performance is affected by the constant acquisition of viral mutations due to the inherent high error rate of virus RNA-dependent RNA polymerase (RdRp) and the existence of a highly variable receptor-binding motif in the spike (S) protein.^11–13^ We have previously noted that the B.1.351 (Beta) variant significantly reduces the neutralizing geometric mean antibody titers (GMT) in recipients^14^ of mRNA and inactivated vaccines and may cause breakthrough infections.^15^ The reduction in neutralization activity has raised concerns about vaccine efficacy. Thus, rapid virus sequence surveillance (e.g. the identification of E484 mutations in new SARS-CoV-2 variants^16^) and vaccine updates are crucial.

**Fig. 1:**
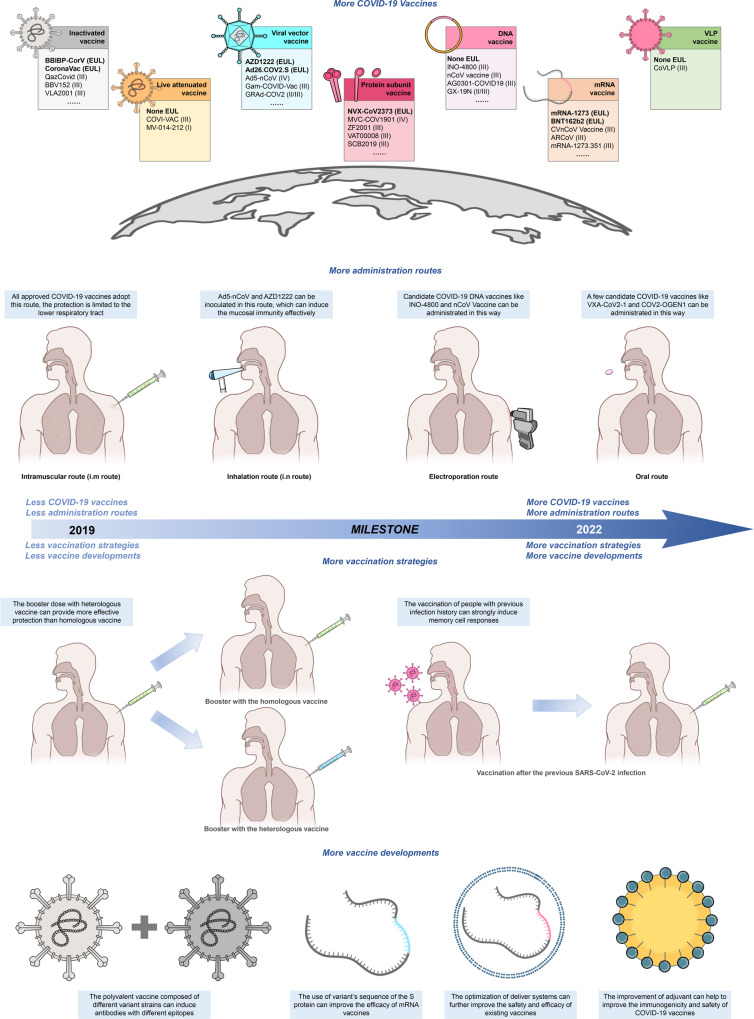
The milestones of COVID-19 vaccine development. With the maturity of vaccine platforms, more and more COVID-19 vaccines have entered clinical trials and been approved for emergency use in many countries. However, the appearance of VOCs has brought great challenges to existing COVID-19 vaccines. By changing the administration route, the protection provided by vaccines can be enhanced, and more vaccination strategies are applied to cope with VOCs. In addition, more vaccine development methods are applied, such as developing polyvalent vaccines and improving adjuvant and delivery systems. These enormous changes form a milestone in the COVID-19 vaccine progress compared with post-years

This review systematically introduces the existing COVID-19 vaccine platforms, analyzes the advantages and disadvantages of the vaccine routes, and compares the efficacy and safety of various vaccines, including the possible complications and different protective efficacies in special populations. Moreover, given the continuous mutation of SARS-CoV-2, we analyze the neutralization activities of various vaccines according to the latest research and propose ideas to improve and optimize existing vaccines, including changing the administration route, adopting more vaccination strategies, and applying more vaccine development methods (Fig. 1).

## Vaccine-induced immunity

The immune response elicited by the body after vaccination is termed active immunity or acquired immunity. In this process, the immune system is activated. CD4^+^ T cells depend on antigen peptide (AP)-MHC (major histocompatibility complex) class II molecular complex to differentiate into helper T cells (Th cells). CD8^+^ T cells depend on AP-MHC class I molecular complex and differentiate into cytotoxic T lymphocytes (CTL). B cells are activated with the help of Th cells to produce antibodies. After antigen stimulation, B and T cells form corresponding memory cells to protect the body from invading by the same pathogen, typically for several years. The development of COVID-19 vaccines is mainly based on seven platforms, which can be classified into three modes according to the antigen category.^17,18^ The first mode is based on the protein produced in vitro, including inactivated vaccines (inactivated SARS-CoV-2), VLP vaccines (virus particles without nucleic acid), and subunit vaccines (S protein or receptor-binding domain (RBD) expressed in vitro). The second model is based on the antigen gene expressed in vivo, including viral vector vaccines (using replication-defective engineered viruses carrying the mRNA of S protein or RBD), DNA vaccines (DNA sequences of S protein or RBD), and mRNA vaccines (RNA sequences of S protein or RBD). The third mode is the live-attenuated vaccine. These vaccines can induce neutralizing antibodies to protect recipients from viral invasion. Moreover, some mRNA and viral vector vaccines can induce Th1 cell responses^19,20^ and persistent human germinal center responses,^21,22^ which provide more efficient protection. In addition, memory cells induced by COVID-19 vaccines play an important role in vaccine immunity.^23–25^

### Vaccine-induced Th1 cell response

**Fig. 2:**
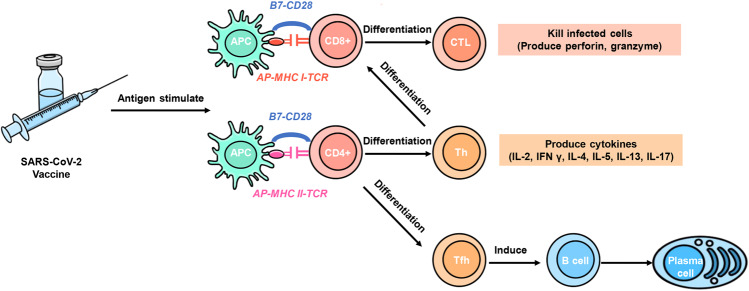
Vaccine-induced Th1 cell response. Some COVID-19 vaccines would induce Th1 cell responses. After recognition of the AP-MHC class II complex and T-cell receptor (TCR), CD4^+^ T cells distributed in peripheral lymphoid organs can differentiate into Th1 cells, which secrete various cytokines, such as interleukin 2 (IL-2), and simultaneously upregulate the expression of related receptors (IL-2R). Through IL-2 and IL-2R, T-cell proliferation and CD8^+^ T-cell activation are promoted, CD8^+^ T-cell can differentiate into cytotoxic T lymphocytes (CTLs) through the activation, producing perforin and other cytokines, which may improve the efficacy of vaccines

ChAdOx1 nCoV-19 (AZD1222, viral vector vaccine), NVX-CoV2373 (protein subunit vaccine), mRNA-1273(mRNA vaccine), BNT162 (including BNT162b1 and BNT162b2, mRNA vaccine), and other COVID-19-candidate vaccines were reported to induce Th1 cell responses.^19,26–28^ After recognition of the AP-MHC class II complex and T-cell receptor (TCR), CD4^+^ T cells distributed in peripheral lymphoid organs can differentiate into Th1 cells, which secrete various cytokines, such as interleukin 2 (IL-2), and simultaneously upregulate the expression of related receptors (IL-2R). After IL-2 binds to IL-2R, T-cell proliferation and CD8^+^ T-cell activation are promoted. Both CD4^+^ and CD8^+^ T-cell responses have been observed in Ad26.COV-2-S recipients.^29,30^ The activated CD8^+^ T cells differentiate into CTLs to further induce cellular immunity. In addition, Th1 cells can secrete interferon-gamma (IFN-γ) and tumor necrosis factor-alpha (TNF-α).^31^ The former also induces the differentiation of CD4^+^ T cells and enhances the intensity of the immune response (Fig. 2).

When the effector cells (Th cells and CTLs) clear the antigen, the signal maintaining the survival and proliferation of T cells no longer exists, the cell responses are reduced, and the immune system returns to homeostasis. However, antigen-specific memory T cells are crucial for long-term protection, typically formed during T-cell-mediated immunity.^23^

### Vaccine-induced germinal center response and humoral immune regulation

**Fig. 3:**
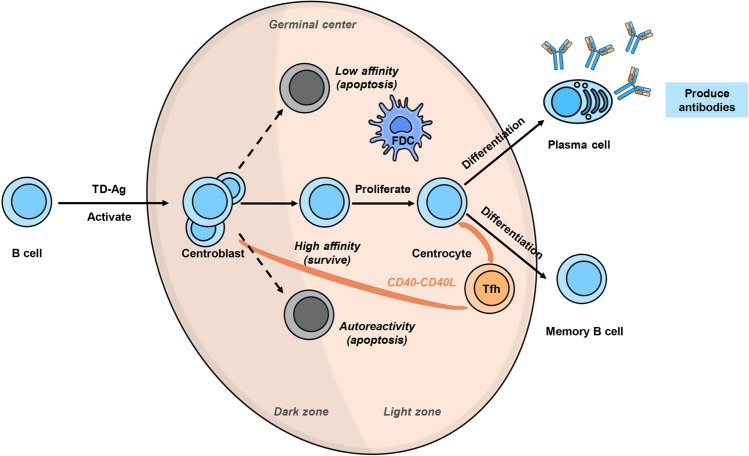
Vaccine-induced germinal center response. Some COVID-19 vaccines would induce a germinal center response. Upon the interaction of T cells and B cells, some activated Th cells move to the lymphatic follicles and then differentiate into Tfh cells. Activated B cells proliferate and divide in lymphatic follicles to form the germinal center. With the help of Tfh cells, high-frequency point mutations occur in the variable region of the antibody gene of GC B cells, and antibody category transformation occurs, finally forming memory B cells and plasma cells, which can produce high-affinity antibodies

In addition to T-cell responses, follicular helper T cells (Tfh cells) induced by mRNA vaccines can trigger effective SARS-CoV-2 antigen-specific germinal center B-cell (GC B-cell) responses (Fig. 3).^21,22,32^ Upon the interaction of T cells and B cells, some activated Th cells move to the lymphatic follicles and then differentiate into Tfh cells. Activated B cells proliferate and divide in lymphatic follicles to form the germinal center. With the help of Tfh cells, high-frequency point mutations occur in the variable region of the antibody gene of GC B cells, and antibody category transformation occurs, finally forming memory B cells and plasma cells, which can produce high-affinity antibodies. In one study, the GC B-cell response of BALB/c mice peaks between 7 and 14 days after the injection of the mRNA vaccine based on full-length S protein. However, the ability of the RBD-based mRNA vaccine to induce GC B-cell response was poor, indicating that the full-length S protein may play an important role in vaccine-induced GC B-cell response.^22^ In addition, a strong SARS-CoV-2 S protein-binding GC B-cell response was detected in lymph node fine-needle aspirates of BNT162b2 (based on full-length S protein) vaccine recipients. The GC B-cell response was detected after the first dose and greatly enhanced after the second dose.^21^

The continuous existence of GC B cells is the premise for inducing long-lived plasma cells.^33^ GC B cells that are not transformed into plasma cells will form memory B cells, and memory B cells are activated rapidly with the help of memory Th cells when encountering the same antigen and then produce plenty of antigen-specific antibodies. It can be concluded that the sustained GC B-cell response induced by the vaccine can secrete potent and persistent neutralizing antibodies and trigger strong humoral immunity.^21^

### COVID-19 vaccine-induced memory cell responses

**Fig. 4:**
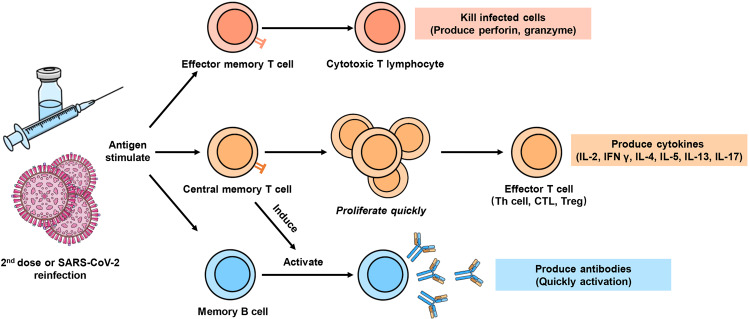
Vaccine-induced memory cell response. In the Th1 and GC B-cell processes, antigen-specific memory T cells and memory B cells are usually formed. Unlike initial T-cell activation, the activation of memory T cells no longer depends on antigen-presenting cells and can induce a stronger immune response. Most memory B cells enter the blood to participate in recycling and are rapidly activated to produce potent antibodies upon encountering the same antigen

The COVID-19 vaccine-induced memory cell responses can induce Th1 and sustained germinal center responses, triggering strong cellular and humoral immunity. In this process, antigen-specific memory T cells and B cells are usually formed, significant for long-term protection (Fig. 4).^23^ Unlike initial T-cell activation, the activation of memory T cells no longer depends on antigen-presenting cells and can induce a stronger immune response. Most memory B cells enter the blood to participate in recycling and are rapidly activated to produce potent antibodies upon encountering the same antigen. The mRNA-1273 and BNT162b2 induced higher-level production of antibodies and stronger memory B-cell response.^24^ Moreover, memory B cells could also be detected in patients who have recovered from COVID-19, and a single dose of mRNA vaccine can induce the memory B-cell response to reach the peak in these patients,^24,34^ indicating that both previous infection and vaccination can induce memory cell responses.

## Existing vaccine platforms for COVID-19 vaccines

**Fig. 5:**
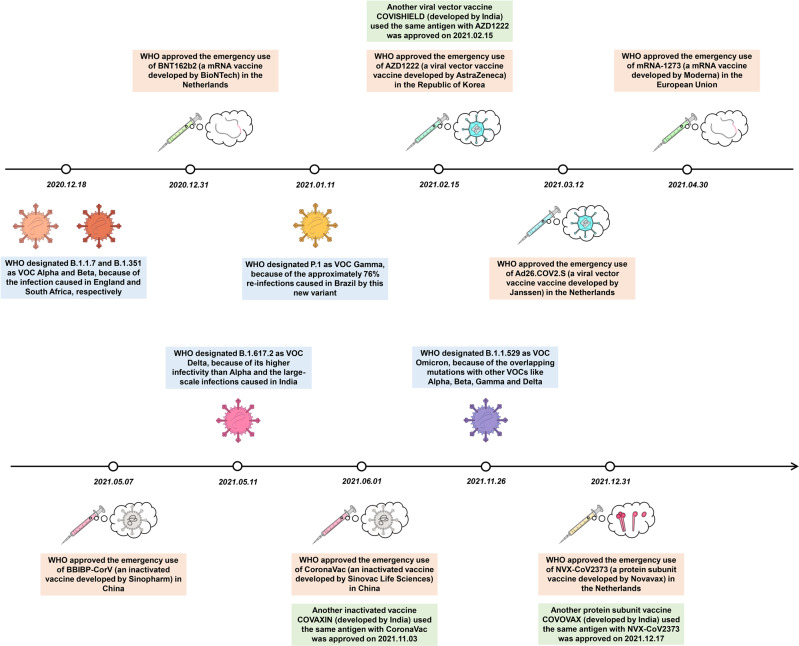
A timeline of critical events in the COVID-19 vaccine development progress. WHO has approved the emergency use of ten vaccines (including three India vaccines, COVISHIELD, COVAXIN, and COVOVAX). Vaccination plays a critical role in protecting people from SARS-CoV-2 infections. However, the appearance of VOCs brought big challenges to the efficacy of approved COVID-19 vaccines. These events were summarized and displayed in the form of a timeline

According to WHO data released on March 28, 2022, 153 vaccines have been approved for clinical trials, and 196 vaccines are in preclinical trials. These vaccines mainly include inactivated vaccines (accounting for 14% of the total), live attenuated vaccines (1%), viral vector vaccines (replication and non-replication; 17% of the total), RNA vaccines (18%), DNA vaccines (11%), protein subunit vaccines (34%), and VLP vaccines (4%) (https://www.who.int/publications/m/item/draft-landscape-of-covid-19-candidate-vaccines). As of March 28, 2022, a total of ten vaccines (including three India vaccines), including inactivated vaccines, viral vector vaccines, mRNA vaccines, and protein subunit vaccines, have been approved for emergency use by WHO (Fig. 5) (https://extranet.who.int/pqweb/vaccines/vaccinescovid-19-vaccine-eul-issued). The features, advantages, and disadvantages of different COVID-19 vaccines are shown in Tables 1, 2.

**Table 1:** The features of various COVID-19 vaccine platforms

Vaccine platforms	Vaccine component	Mechanism of induction antibody	Name of vaccine approved by WHO (vaccine manufacturer)	Number of clinical trials on phase III and Phase IV of vaccines not yet approved by WHO (Trial identifier) (Until March 28, 2022)
Inactivated vaccine	Entire viruses are cultured in vitro and inactivated by chemical reagents.^35^	The entire virus as an immunogen induces a wider range of antibodies against different epitopes.^17^	BBIBP-CorV (Sinopharm) CoronaVac (Sinovac Biotech) COVAXIN (Bharat Biotech International)	Twelve. (WIBP- CorV: NCT04885764, ChiCTR2000034780, NCT05065892; Inactivated SARS-CoV-2 vaccine (Vero cell) manufactured by IMBCAMS: NCT04659239; QazCovid-in®: NCT04691908; BBV152: NCT04918797, NCT04641481; KCONVAC: NCT04852705; VLA2001: NCT04864561; TURKOVAC: NCT04942405; CovIran-Barkat: IRCT20201202049567N3; KD-414: jRCT2071210081)
Live attenuated vaccine	The virus is obtained by reverse genetics or adaptation.^17^	a) The retained viral amino acid sequences induce extensive responses, including innate, humoral, and cellular immunity.^3^ b) Induce mucosal immunity through nasal inhalation to protect the upper respiratory tract.^3^	Not yet	One. (COVI-VAC: ISRCTN15779782)
Viral vector vaccine	Engineered viruses with replication-attenuated carrying genetic material of viral proteins or polypeptides.^35^	Viral vector vaccines can induce Th1 cell responses, thus inducing strong protective effects.^29,50^	AZD1222(AstraZeneca-University of Oxford) Ad26.COV-2-S (Johnson & Johnson) COVISHIELD (Serum Institute of India)	Nine. (Ad5-nCoV: NCT04526990, NCT04892459; Ad5-nCoV-IH: NCT05124561; Gam-COVID-Vac: NCT04530396; GRAd-COV-2: NCT04791423; DelNS1-2019-nCoV-RBD-OPT1: ChiCTR2100051391; IIBR-100: NCT04990466; AZD2816: NCT04973449; BBV154: CTRI/2022/02/040065)
Protein subunit vaccine	Cell-expressing systems express viral proteins or peptides systemically.^17^	Induce Th1 cell responses.^260^	NVX-CoV2373 (Novavax) COVOVAX (Serum Institute of India)	Twenty-three. (ZF2001: ChiCTR2100050849; VAT00008: ACTR202011523101903; RBD Recombinant SARS-CoV-2 vaccine: NCT04887207; SCB-2019: NCT05012787; SpikoGen: NCT05005559, IRCT20150303021315N24; aCoV2: NCT04806529; MVC-COV1901: NCT05011526, NCT05079633; FINLAY-FR-2: RPCEC00000354; EpiVacCorona: NCT04780035; Recombinant COVID-19 vaccine (Sf9 cells): NCT04904471; UB-612: NCT04683224; CIGB-66: RPCEC00000359; BECOV2A: CTRI/2021/08/036074; Nanocovax: NCT04922788; S-268019: NCT05212948; GBP510: NCT05007951; Razi Cov Pars: IRCT20210206050259N3; ReCOV: NCT05084989; V-01: NCT05096832; Noora Vaccine: IRCT20210620051639N3; SCTV01C: NCT05043311)
DNA vaccine	Viral antigens encoded by a recombinant plasmid.^17^	Induce neutralizing antibodies.^17^	Not yet	Five. (INO-4800+electroporation: NCT04642638, ISRCTN15779782; AG0301-COVID-19: NCT04655625; nCov vaccine: CTRI/2020/07/026352; GX-19N: NCT05067946)
mRNA vaccine	mRNA encapsulated by vectors, viral proteins, or polypeptides.^17^	Induce strong Th1 cell responses, GC B-cell responses and simultaneously produce long-lived plasma cells and memory cells to elicit neutralizing antibodies.^21,24^	BNT162b2 (Pfizer-BioNTech) mRNA-1273 (Moderna)	Nine. (CVnCoV: NCT04652102, NCT04674189; ARCoV: NCT04847102; mRNA-1273.351: EUCTR2021-000930-32; DS-5670a: jRCT2071210106; mRNA-1273.211: NCT04927065; ARCT-154 mRNA Vaccine: NCT05012943, ISRCTN15779782; mRNA-1273.529-Booster: NCT05249829)
VLP vaccine	Noninfectious particles consist of viral structural proteins and viral polypeptides.^17^	Antigens loading on the protein particles induce neutralizing antibodies against immune epitopes.	Not yet	One. (CoVLP: NCT04636697)

*WHO* World Health Organization, *VLP* virus-like particle

**Table 2:** Comparison of advantages and disadvantages of the COVID-19 vaccine

Platform	Advantages	Disadvantages
COVID-19 inactivated vaccine	1. Inactivated vaccines use the entire virus as an immunogen, inducing an immune response and producing antibodies against S protein, N protein, E protein, and other regions.^17^ 2. Compared with vaccines based on the SARS-CoV-2 S protein or partial protein fragments, inactivated vaccines can induce a wider range of antibodies against more epitopes.^17^ 3. The research and development route of inactivated vaccines is relatively complete. 4. The overall adverse reaction rate of inactivated vaccines in clinical trials is low. No deaths have been reported in clinical trials, which indicates that inactivated vaccines have good safety.^38–40^	1. Due to living viruses, the production must be carried out in a biosafety level-3 laboratory or higher biosafety level.^3^ 2. The production of inactivated vaccines is limited by cell activity and viral productivity.^35^
COVID-19 live attenuated vaccines	1. Through the CPD-based method allows most viral amino acid sequences to be retained and induce extensive responses, including innate, humoral, and cellular immunity against viral structural and nonstructural proteins in the recipient.^3,43^ 2. The extensive response is unlikely to diminish in efficacy due to antigen drift.^3^ 3. Live attenuated vaccines can induce mucosal immunity through nasal inhalation to protect the upper respiratory tract. In contrast, other vaccines are usually administered intramuscularly and only protect the lower respiratory tract. 4. The virus replicates and proliferates in the vaccinator, which may simultaneously induce humoral and cellular immunity, resulting in a multidirectional antiviral effect.^3,43^	1. After weakening the virulence gene of the virus, virulence may be restored during replication and proliferation in the host. Thus, the reverse genetic method remains challenging. 2. Loss of efficacy and reproductive potential of viruses during vaccine production poses a significant challenge.^17^ 3. There may be safety problems in producing live attenuated vaccines.
COVID-19 Viral vector vaccines	1. The manufacturing process of viral vector vaccines is relatively safe compared with inactivated vaccines as there is no need to deal with live SARS-CoV-2.^3^ 2. Viral vector vaccines can induce Th1 cell responses, inducing stronger protective effects. The carrier can strengthen immunity and have a good stimulating response to B cells and T cells.^29,50^ 3. The platform has been used in previous vaccines. Therefore, there is rich experience in the preparation of vectors.	1. Adenovirus-based viral vector vaccines can induce complications, especially thrombocytopenia. Thus, it is necessary to pay attention to the platelet levels of the relevant recipients in case of thrombocytopenia.^51,52^ 2. Although adenovirus is not easily neutralized by pre-existing immunity, some individuals with neutralizing antibodies against several adenoviruses (including Ad5) in the serum may reduce the immunogenicity.^26,54^
COVID-19 Protein subunit vaccine	1. Could induce Th1 cell responses.^31^ 2. NVX-CoV2373 can induce higher titer neutralizing antibodies than inactivated and Ad5 viral vector vaccines.^3^ 3. In the past, protein subunit vaccines have been successfully applied. Moreover, the research and development idea is relatively clear.	1. The S protein has a large molecular weight, and the expression efficiency of the S protein is relatively low compared with that of RBD. 2. Although the RBD has a small molecular weight and is easy to express, it lacks other immune epitopes on the S protein thus is prone to antigen drift.^3^
COVID-19 DNA vaccines	1. Compared with mRNA vaccines, DNA vaccines have higher stability and can be stored for a long time.^65^ 2. *Escherichia coli* can be used to prepare plasmids with high stability. Therefore, the production risk of DNA vaccines is relatively low.^3^	1. The immunogenicity of the DNA vaccine is low.^3^ 2. Different injection methods will affect the vaccine’s efficacy, such as intramuscular or electroporation injection.^3^
COVID-19 mRNA vaccines	1. Both BNT162b1 and BNT162b2 vaccines transmit the genetic information of the antigen rather than the antigen itself.^3^ Therefore, mRNA vaccines only need to synthesize corresponding RNA/DNA of viral proteins, improving production speed.^35^ 2. The mRNA vaccines can induce strong Th1 cell responses and GC B-cell responses and produce long-lived plasma cells and memory B cells, which can continuously elicit SARS-CoV-2 neutralizing antibodies.^21,24^ 3. The mRNA vaccines can be modified directly on the original sequence to facilitate a timely update.	1. The mRNA vaccines may cause complications, especially myocarditis.^54,74,75^ 2. Due to the instability of mRNA, the mRNA vaccines need to be stored at a lower temperature, which puts forward certain requirements for the storage environment of the inoculation unit.
COVID-19 VLP vaccines	1. VLP vaccines do not contain viral genomes. Thus, they are not infectious.^74^ 2. Plant-based VLP vaccines have the potentiality of oral delivery vaccines.^65^ 3. By loading a variety of antigens, such as the RBD from different variants on the protein particles, neutralizing antibodies against multi-immune epitopes can be induced to improve the neutralizing activity against SARS-CoV-2 variants.	1. The manufacturing process of the VLP vaccine is more complex. What’s more, there is no relevant data published for human clinical trials. 2. The VLP vaccines are loaded with multiple proteins simultaneously, and the degree of immune response caused by it is not clear.^65^

*S protein* spike protein, *N protein* nucleocapsid protein, *E protein* envelope protein, *Ad5* adenovirus type-5, *CPD* codon pair deoptimization, *RBD* receptor-binding domain

### COVID-19 inactivated vaccines

Inactivated vaccines are produced by inactivating the in vitro cultured viruses using chemical reagents.^35^ The vaccine can maintain the integrity of virus particles as immunogens.^17^ Wang et al. introduced the manufacturing process of the SARS-CoV-2 inactivated vaccine. In this process, SARS-CoV-2 from throat swabs of COVID-19 patients were used to infect Vero cells, and the HB02 strain with the strongest replication ability was selected from three isolated strains (HB02, CQ01, and QD01). After purification, the P1 library was obtained by subculturing in Vero cells with adaptive culturing, subculturing, and amplification. The seventh-generation virus, BJ-P-0207, was selected as the original strain of the COVID-19 inactivated vaccine,^36,37^ and then β-propiolactone was used to inactivate the virus.^37^

An advantage of inactivated vaccines is using the entire virus as an immunogen. Compared with vaccines based on the SARS-CoV-2 S protein or partial protein fragments, such as RBD, inactivated vaccines can induce a wider range of antibodies against more epitopes.^17^ In addition, the overall adverse reaction rate of inactivated vaccines in clinical trials is low, and no deaths have been reported in clinical trials, indicating their good safety.^38–40^ However, the production of inactivated vaccines are limited because the production of such vaccines must be carried out in biosafety level-3 laboratory or higher biosafety level.^3^

The BBIBP-CorV and CoronaVac inactivated vaccines approved by WHO are independently developed in China. A total of 21 candidate COVID-19 inactivated vaccines have been approved for clinical trials as of March 28, 2022 (https://www.who.int/publications/m/item/draft-landscape-of-COVID-19-candidate-vaccines).

### COVID-19 live attenuated vaccines

Live attenuated vaccines are based on the virus obtained by reverse genetics or adaptation to reduce virulence and are used as non-pathogenic or weakly pathogenic antigens.^17^ Currently, the main manufacturing processes include codon pair deoptimization (CPD) and virulence gene knockout.^3,41,42^ Wang et al. and Trimpert et al. reported the CPD-based methods to modify SARS-CoV-2 genes genetically. In their studies, amino acid (aa) 283 deletion was introduced into the S protein, and the furin site was also deleted to attenuate the virulence of the virus but retain its replication ability.^43,44^

Through the CPD-based method, most of the viral amino acid sequences can be retained and induce extensive responses, including innate, humoral, and cellular immunity against viral structural and nonstructural proteins in the recipient.^3,43^ The extensive response is unlikely to diminish in efficacy due to antigen drift. In addition, live attenuated vaccines can induce mucosal immunity through nasal inhalation to protect the upper respiratory tract.^3^ In contrast, other types of vaccines, such as inactivated and mRNA vaccines, are usually administered intramuscularly and only protect the lower respiratory tract. However, after weakening the virulence gene of the virus, virulence may be restored during replication and proliferation in the host. Thus, the reverse genetic method remains challenging.

Currently, there is no WHO-approved COVID-19 live attenuated vaccine for emergency use. Two candidate COVID-19 live attenuated vaccines, COVI-VAC and MV-014-212, have been approved for clinical trials as of March 28, 2022 (https://www.who.int/publications/m/item/draft-landscape-of-COVID-19-candidate-vaccines).

### COVID-19 viral vector vaccines

Viral vector vaccines are based on replication-attenuated engineered viruses carrying genetic material of viral proteins or polypeptides.^35^ The particular antigen is produced by host cells after immune transduction.^17^ Zhu et al. reported the manufacturing process of a viral vector vaccine based on human adenovirus type-5 (Ad5). In this process, the signal peptide gene and optimized full-length S protein gene based on the Wuhan-Hu-1 strain were introduced into a human Ad5 engineering virus with E1 and E3 gene deletions to produce a vector expressing S protein.^45^ A recombinant chimpanzee Ad25 vector expressing full-length S protein was used to prepare the ChAdOx1 nCoV-19 vaccine.^46^ Recombinant vectors based on the combination of human Ad5 and Ad26 were also used to prepare the Sputnik V vaccine.^47,48^ In addition, the Ad26.COV-2-S vaccine developed by Janssen is based on the S protein modified by the Ad26 expression gene, with the deletion of the furin site and the introduction of aa986-987 mutations.^48^ Besides adenovirus, vesicular stomatitis virus can also be modified and used to produce the COVID-19 vaccine, inducing a stronger humoral immune response via intranasal and intramuscular routes.^49^

Except for inactivated vaccines and partially attenuated vaccines, there is no need to deal with live SARS-CoV-2 in manufacturing other types of vaccines (e.g., viral vector, protein subunit, mRNA, DNA, and VLP vaccines), so the manufacturing process of these vaccines is relatively safe.^3^ In addition, viral vector vaccines can induce Th1 cell responses,^29,50^ thus inducing strong protective effects. However, adenovirus-based viral vector vaccines can induce complications, especially thrombocytopenia. Thus, it is necessary to pay attention to the platelet levels of the relevant recipients in case of thrombocytopenia.^51,52^ Although adenovirus is not easily neutralized by pre-existing immunity, the pre-existing Ad5 antibodies (46.4, 80, 78, 67, 64, 60, 45% and less than 30% of the population with neutralizing antibodies titers for Ad5 of >1:200 in China, India, Kenya, Thailand, Uganda, South Africa, Sierra Leone, and America, respectively,^26,53^) these pre-existing adenoviruses antibodies in the serum may reduce the immunogenicity of such vaccines. Thus an additional flexible dose might be needed as a solution.^26,54^

The WHO has approved two viral vector vaccines (Ad26.COV-2-S and AZD1222). As of March 28, 2022, 25 candidates’ clinical trials for COVID-19 viral vector vaccines have been approved, with four using replicating vectors and 21 using non-replicating vectors. Moreover, 3 viral vectors (a type of nonreplicable vector and two types of replicable vectors) + antigen-presenting cells and a vaccine based on the bacterial antigen-spore expression vector are also approved for clinical trials (https://www.who.int/publications/m/item/draft-landscape-of-covid-19-candidate-vaccines).

### COVID-19 protein subunit vaccine

Protein subunit vaccines are based on systemically expressed viral proteins or peptides using various cell-expressing systems, such as bacteria, yeasts, insects, and mammalian cells (such as human embryonic kidney cells).^17,35,55–57^ These vaccines can be divided into recombinant S protein and RBD vaccines.^3^ The ZF2001 vaccine adopts the dimer form of the S protein RBD of SARS-CoV-2 as an antigen.^58^ Another subunit vaccine (NVX-CoV2373) adopts a full-length S protein with a pre-fusion conformation containing a furin site mutation, and the modified S protein was produced by the Sf9 insect cell expression system. The S protein with a pre-fusion conformation is usually metastable and easily transformed into the post-fusion conformation. The pre-fusion conformation can be stabilized by mutating two residues (K986 and V987) to proline.^17,59^ In addition, a recombinant vaccine comprising residues 319–545 of the RBD was manufactured using insect cells and a baculovirus expression system, and the purity of the recombinant protein was more than 98% by adding a GP67 signal peptide in the expression system.^60^

The protein subunit can also induce Th1 cell responses.^31^ In addition, NVX-CoV2373 can induce higher titer neutralizing antibodies than inactivated and Ad5 viral vector vaccines.^3^ However, the S protein has a large molecular weight, and the expression efficiency of the S protein is relatively low compared with that of RBD. Although the RBD has a small molecular weight and is easy to express, it lacks other immune epitopes on the S protein and thus is prone to antigen drift.^3^

For emergency use, the WHO has authorized only one COVID-19 protein subunit vaccine (NVX-CoV2373). Furthermore, 51 candidate COVID-19 protein subunit vaccines were approved for clinical trials on March 28, 2022 (https://www.who.int/publications/m/item/draft-landscape-of-covid-19-candidate-vaccines).

### COVID-19 DNA vaccines

DNA vaccines are based on viral antigens encoded by a recombinant plasmid. Viral proteins or polypeptides are produced by transcription and translation processes in host cells.^17^ Smith et al. synthesized the INO-4800 COVID-19 DNA vaccine based on a previously prepared MERS-CoV vaccine.^61^ The main steps are as follows: (1) acquisition of the S protein sequence from GISAID; (2) addition of the N-terminal IgE leading sequence; (3) optimization of the IgE-Spike sequence with algorithms to enhance its expression and immunogenicity and synthesize the optimized sequence; (4) ligation of the fragment into the expression vector pGX0001 after digestion.^62,63^ Brocato et al. constructed the DNA encoding SARS-CoV-2 S protein into the pWRG skeleton plasmid by cloning the gene with optimized human codons, and this skeleton plasmid was used to produce a DNA vaccine against hantavirus.^64^

Compared with mRNA vaccines, DNA vaccines have higher stability and can be stored for a long time.^65^ *Escherichia coli* can be used to prepare plasmids with high stability.^3^ However, the immunogenicity of the DNA vaccine is low. Furthermore, different injection methods, such as intramuscular or electroporation injection, also affect the vaccine’s efficacy.^3^

There is no COVID-19 DNA vaccine authorized by the WHO for emergency use. Sixteen candidate COVID-19 DNA vaccines have been approved for clinical trials on March 28, 2022 (https://www.who.int/publications/m/item/draft-landscape-of-covid-19-candidate-vaccines).

### COVID-19 mRNA vaccines

mRNA vaccines are based on mRNA encapsulated by vectors (usually lipid nanoparticles), viral proteins, or polypeptides produced during the translation process in the host cells.^17,35^ In addition to mRNA itself, the 5′ Cap and 3′ Poly (A) also play important roles in regulating the efficiency and stability of translation.^66,67^ At present, mRNA vaccines usually adopt the Cap 1 structure (m^7^GpppN_1_mp, with an additional 2′ methylated hydroxyl compared with Cap 0), improving translation efficiency.^66^ There are two ways of mRNA tailing: use traditional polyadenylate tails to add the 3′ tail of poly (A) or design the DNA template with a proper length of poly (A), and the latter can obtain a length-controlled poly (A) tail.^67,68^ Corbett et al. introduced a manufacturing process for the mRNA-1273 vaccine. The optimized mRNA encoding SARS-CoV-2 S-2P protein with stable pre-fusion conformation was synthesized (2 P represents double proline mutations of the K986 and V987 residues mentioned above). The synthesized mRNA sequence was purified by oligo-dT affinity purification, and encapsulated in lipid nanoparticles.^69^ The BNT162b2 vaccine also adopts a similar mRNA encoding S-2P,^17,70^ whereas the BNT162b1 vaccine adopts the mRNA encoding RBD and fuses the trimer domain of T4 fibrin to the C-terminus. Furthermore, a proper delivery system like LNP can protect mRNA against the degradation of nuclease^71^ and further enhance the efficacy of mRNA vaccines. The capsulation of mRNA with LNP can effectively transfer mRNA into cells and induce a strong immune response; thus is widely used in most mRNA vaccines, including BNT162b2 and mRNA-1273.^71,72^ In addition, other delivery systems like lipopolyplexes, polymer nanoparticles, cationic polypeptides, and polysaccharide particles also provide unlimited possibilities for the improvement of mRNA vaccine .^72,73^

The mechanism of mRNA vaccine-induced immunity is similar to that of the DNA vaccines. Both BNT162b1 and BNT162b2 vaccines transmit the genetic information of the antigen rather than the antigen itself,^3^ so they only need to synthesize the corresponding RNA of viral proteins, improving the production speed.^35^ In addition, mRNA vaccines can induce strong Th1 cell responses and GC B-cell responses and simultaneously produce long-lived plasma cells and memory cells, continuously eliciting SARS-CoV-2 neutralizing antibodies.^21,24^ However, mRNA vaccines may cause complications, especially myocarditis,^54,74,75^, and have a higher storage requirement due to the instability of mRNA.^3^

The WHO has approved two types of mRNA vaccines: mRNA-1273 and BNT162b2, and a total of 28 candidate COVID-19 mRNA vaccines have been approved for clinical trials as of March 28, 2022 (https://www.who.int/publications/m/item/draft-landscape-of-covid-19-candidate-vaccines).

### COVID-19 VLP vaccines

VLP vaccines are based on noninfectious particles consisting of in vitro-expressed viral structural proteins and decorated viral polypeptides on the surface.^74^ Tan et al. used Spy Tag technology to modify the SARS-CoV-2 RBD on the surface of protein particles by forming covalent iso-peptide bonds based on the previous protein nanoparticle platform and obtained an RBD-Spy VLP.^76^ Moreover, a self-assembled VLP vaccine based on the expression of modified full-length S proteins, including R667G, R668S, R670S, K971P, and V972P mutations, has also been developed using a plant expression system.^77^

VLP vaccines do not contain viral genomes, and plant-based VLP vaccines have the potential of oral delivery vaccines.^65^ By loading a variety of antigens, such as the RBD from different variants on the protein particles, neutralizing antibodies against multi-immune epitopes can be induced to improve the neutralizing activity against SARS-CoV-2 variants. However, the manufacturing process of the VLP vaccine is more complex, and no relevant data was published for human clinical trials.

There is no COVID-19 VLP vaccine authorized by the WHO for emergency use. Six candidates' COVID-19 VLP vaccines have been approved for clinical trials as of March 28, 2022 (https://www.who.int/publications/m/item/draft-landscape-of-covid-19-candidate-vaccines).

## Efficacy of covid-19 vaccines

### Animal studies of COVID-19 vaccines approved by the WHO

**Fig. 6:**
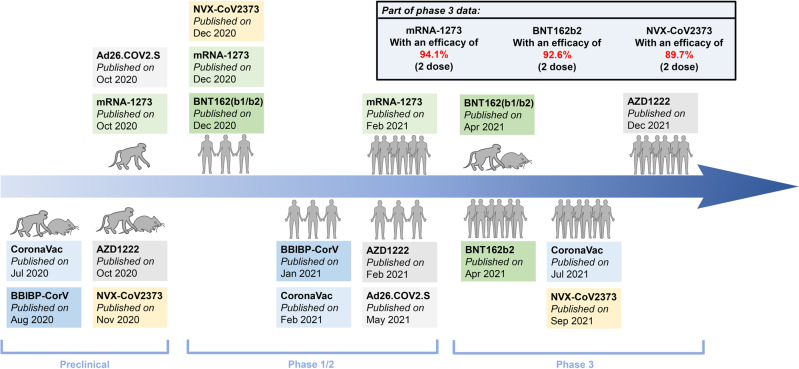
A timeline of the preclinical and clinical trials of approved COVID-19 vaccines. Preclinical and clinical trials play important roles in evaluating the safety and protective efficacy of COVID-19 vaccines. The information of preclinical to clinical trials of several WHO-approved COVID-19 vaccines are provided in the form of a timeline, and partial Phase III clinical trials’ data were also displayed to show the total efficacy

Several SARS-CoV-2 animal models have been developed, including mice expressing human ACE2,^78–80^ SARS-CoV-2-adaptive mouse,^81,82^ ferret,^83^ hamster,^84,85^ and NHP models.^86–88^ Although mice can be infected with SARS-CoV-2 by transferring the human ACE2 gene or designing a virus-adapted mouse, no mouse model can simulate all the characteristics of human COVID-19, especially pulmonary vascular disease, hyperinflammatory syndrome, observed in adults and children, respectively.^10^ The hamster model can simulate serious COVID-19 diseases. Syrian hamsters show mild to severe symptoms 1–2 days after nasal infection,^89,90^ and progressive weight loss and dyspnea. The NHP model can reflect mild-to-moderate SARS-CoV-2 infection and can be used to test many candidate vaccines. However, due to different adjuvants and vaccine dosages, the use of serum-neutralizing antibody titer as a direct basis for comparing the efficacy of different vaccines is still limited. In addition, different analytical methods, such as 50% plaque reduction neutralization test (PRNT_50_), 80% plaque reduction neutralization test (PRNT_80_), and enzyme-linked immunosorbent assay (ELISA), may also affect the final experimental results. These data can objectively show the efficacy of each vaccine. Here, we summarize the immunogenicity, neutralizing activity, and cell response data from animal experiments for the BBIBP-CorV, CoronaVac, AZD1222, Ad26.COV-2-S, NVX-CoV2373, mRNA-1273, and BNT162b2 vaccines (Fig. 6).

Immunogenicity testing of BBIBP-CorV was performed in BALB/c mice, rabbits, and guinea pigs.^36^ The animals were classified into three groups according to the doses: high (8 μg), medium (4 μg), and low (2 μg). All dosages produced good immunogenicity, and the serum conversion rate reached 100% on day 21 after immunization. In different dosage groups of BALB/c mice, the immunogenicity of the three-dose group was significantly higher than the two- and single-dose groups. In the NHP experiment, after vaccination, the neutralizing GMTs in rhesus monkeys were 1:860 in the high-dose group and 1:512 in the low-dose group, respectively, indicating BBIBP-CorV can effectively prevent SARS-CoV-2 infection in rhesus monkeys.

The PiCoVacc inactivated vaccine, also known as CoronaVac, is highly immunogenic in BALB/c mice.^37^ After the injection of PiCoVacc, the serum S-specific antibody level of mice was ten times higher than that of convalescent serum obtained from COVID-19 patients. PiCoVacc could induce high RBD antibodies, 30 times higher than the induced NTD antibodies. The neutralizing antibody titer in rhesus monkeys was 1:50 in the third week after one dose of PiCoVacc, similar to the titers in the convalescent serum of COVID-19 patients. One week after the third dose of PiCoVacc, viral infection was induced through intranasal and organ routes. The viral load of all vaccinated animals decreased significantly 3–7 days after infection, indicating that PiCoVacc played an important anti-SARS-CoV-2 role in the NHP model.

Compared with BBIBP-CorV and CoronaVac, viral vector vaccines and mRNA vaccines can simultaneously induce T-cell responses,^46,48,69,70^ mainly a Th1 cell response, while Th2 responses are related to vaccine-induced respiratory diseases, and were not detected. Viral-specific neutralizing antibodies were detected in all BALB/c mice following inoculation with ChAdOx1 nCoV-19 (AZD1222). On day 14, after the first or second dose, the neutralizing antibody titers in rhesus monkey serum were 1:5 to 1:40 (single dose) and 1:10 to 1:160 (two doses). In addition, cytokines, including IL-4, IL-5, and IL-13, in rhesus monkey serum after a single dose or two doses injection were low, indicating the safety of ChAdOx1 nCoV-19 in NHPs.

Another viral vector vaccine, Ad26.COV-2-S (Ad26-S.PP) induced similar neutralizing antibody titers in the NHP model.^48^ RBD-specific neutralizing antibodies were detected in 31 of 32 rhesus monkeys (96.9%) 2 weeks after Ad26-S.PP inoculation and the induced titers were 1:53 to 1:233 (median 1:113) 4 weeks after vaccination. In addition, Ad26-S.PP also induced S-specific IgG and IgA responses in bronchoalveolar lavage (BAL) obtained from rhesus monkeys, indicating that Ad26-S.PP has a protective effect on rhesus monkeys’ upper and lower respiratory tracts. 6 weeks after vaccination, 1.0 × 10^5^ 50% tissue culture infectious dose (TCID_50_) of SARS-CoV-2 was challenged in intranasal and tracheal routes, and 17 of 32 rhesus monkeys inoculated with Ad26-S.PP were completely protected, and no viral RNA was detected in BAL or nasal swabs, indicating that Ad26-S.PP protects the upper and lower respiratory tracts in the NHP model.

Besides Ad26.COV-2-S, another protein subunit vaccine NVX-CoV2373, also showed the protection efficacy of both upper and lower respiratory tracts in the cynomolgus macaque model.^91^ The vaccine induced a remarkable level of anti-S IgG in mice with the titers of 1:84,000-1:139,000 on the 15th day after the single injection.^59^ Meanwhile, NVX-CoV2373 also elicits multifunctional CD4^+^ and CD8^+^ T-cell responses. In the NHP model, the serum neutralizing antibody titers produced after the second dose of 2.5, 5, 25 μg vaccine could achieve 1:17,920-1:23,040 CPE_100_, which was 7.1–10 times higher than those in convalescent serum. SARS-CoV-2 was challenged in the upper and lower respiratory tract routes after NVX-CoV2373 vaccination, and 91.6% (11 in 12) immunized animals were free of infection. No viral RNA was detected in the nasal swabs, indicating the broader protection of NVX-CoV2373.

The mRNA-1273 vaccine is most immunogenic in the NHP model. The GMTs of rhesus monkey serum obtained from injection dosages of 10 and 100 μg were 1:501 and 1:3,481, respectively, which were 12 times and 84 times higher than that of human convalescent serum.^69^ It has been shown that mRNA-1273 induces a strong S-specific neutralizing antibody response. Rhesus monkeys also showed a dose-dependent Th1 cell response after the injection of mRNA-1273, which was similar to the phenomenon observed after the injection of ChAdOx1 nCoV-19. Intranasal and tracheal routes administered all rhesus monkeys 1.0 × 10^6^ TCID_50_ of SARS-CoV-2 in the 4th week after the second dose. Four days after infection, only low-level viral RNA in two of eight animals in the 10-μg-dose group and one of eight in the 100-μg-dose groups could be detected, indicating good antiviral activity of mRNA-1273 in the NHP model.

BNT162b1 and BNT162b2 (especially the former) also showed high immunogenicity in BALB/c mice while lower than mRNA-1273.^70^ On day 28, after single-dose injection, the serum neutralizing antibody titers of mice with BNT162b1 and BNT162b2 reached 1:1056 and 1:296, respectively. Additionally, both vaccines induced high CD4^+^ and CD8^+^ T-cell responses. In the NHP model, the neutralizing antibody titers of rhesus monkey serum obtained from 100 μg-dose 14 days after vaccination with the second dose of BNT162b1 and BNT162b2 were 1:1714 and 1:1689, respectively, which were significantly higher than those in the convalescent serum of COVID-19 patients (1:94). All rhesus monkeys were administered 1.05 × 10^6^ plaque-forming units of SARS-CoV-2 by intranasal and tracheal routes on 41–55 days after the second dose of BNT162b1 or BNT162b2. On the third day after infection, viral RNA was detected in the BAL of two of the six rhesus monkeys injected with BNT162b1. Viral RNA was not detected in BAL of the BNT162b2 injected monkeys at any time point.

mRNA, viral vector, and protein subunit vaccines showed higher induced-antibody titers than inactivated vaccines and could induce Th1 cell responses. These vaccines mainly induced IgG production and showed a protective effect on the upper respiratory tract. However, the Ad26.S-PP and NVX-CoV2373 vaccines exerted a protective effect on both the upper and lower respiratory tracts. In addition, all injection groups showed significant virus clearance ability after the virus challenge, demonstrating the protection provided by these vaccines in NHPs. Furthermore, all experimental animals injected with the vaccine showed no pathological changes in the lungs and normal tissues, providing strong support for follow-up clinical trials.

### Clinical trials of COVID-19 vaccines approved by the WHO

The safety and effectiveness of vaccines are evaluated in preclinical trials. Clinical trials of candidate vaccines can be carried out only after the relevant data meet the standards for such trials. Ten candidate vaccines have been approved for Phase IV clinical trials. They include three inactivated vaccines (BBIBP-CorV, WIBP COVID-19 vaccine, and CoronaVac), three viral vector vaccines (AZD1222, Ad5-nCoV, and Ad26.COV-2-S), one protein subunit vaccine (MVC-COV1901), and three mRNA vaccines (mRNA-1273, BNT162b2, and mRNA-1273.351) (https://www.who.int/publications/m/item/draft-landscape-of-covid-19-candidate-vaccines). Data from Phase I, I/II, II, II/III, and III trials and some data from Phase IV clinical trials have been released (Fig. 6). Here, the neutralization efficacy, adverse reactions, and cell responses, mainly Th1 cell responses of some vaccines in different clinical trial stages, are discussed. Because of the different adjuvants used and different dosages of the vaccines, the titer of serum neutralizing antibodies cannot be used as a direct reflection of neutralization ability. Moreover, different analysis methods also affect the trial results.

#### BBIBP-CorV

Sinopharm announced the results of a randomized, double-blind, placebo-controlled Phase I/II clinical trial of the BBIBP-CorV vaccine (ChiCTR2000032459).^38^ The Phase I and Phase II trials included 192 and 448 healthy aged 18–80 participants, respectively. All participants were negative for serum-specific SARS-CoV-2 IgG or IgM. In the Phase I trial, the vaccine group was injected with 2–8 μg BBIBP-CorV on day 0 and day 28. The control group was injected with two doses of normal saline placebo containing aluminum hydroxide adjuvant. In the Phase II trial, the vaccine group was divided into single-dose (day 0, 8 μg) and two doses (day 0, day 14, 21, 28; 4 μg at each time). In the Phase II trial, on day 28, after the second dose in the two-dose group or after the single dose in the single-dose group, serum neutralizing antibody titers against SARS-CoV-2 were detected based on PRNT_50_. The antibody titer in the single-dose group was 1:14.7, and the titers range of the two-dose group were 1:169.5-1:282.7. The serum titers after two doses on days 0 and 21 were the highest, indicating that two doses of vaccination could induce a higher neutralizing antibody level. In addition, the Phase I trial showed that the serum titer of subjects >60 years old after 28 days of the second dose was less than that of subjects aged 18–59, indicating that the elderly may need higher doses or adjuvants with stronger immunogenicity. None of the subjects in Phase I/II trials displayed severe adverse reactions within 28 days after vaccination. BBIBP-CorV was demonstrated safe for humans. Currently, several Phase IV clinical trials of the vaccine are underway (NCT04863638, NCT05075070, NCT05075083, NCT05104333, NCT05105295, and NCT05104216) (https://clinicaltrials.gov).

Huang et al. showed that the neutralization ability of serum neutralizing antibody induced by both BBIBP-CorV inactivated vaccine and ZF2001 subunit vaccine to the Beta variant was reduced by 1.6 times.^92^ It is worth noting that serum neutralization activity obtained from BBIBP-CorV homologous booster group and BBIBP-CorV/ZF2001 heterologous booster group were increased, while 80% of samples still failed to neutralize B.1.1.529(Omicron) variant.^93^ The results showed that it is necessary to closely monitor the neutralization efficacy of the vaccine against variants, especially those with strong immune escape ability, such as Beta and Omicron, and update the sequence of seed strain in time.^94^

#### CoronaVac

Sinovac conducted several randomized, double-blind, placebo-controlled Phase I/II clinical trials for the CoronaVac vaccine (NCT04551547, NCT04352608, NCT04383574).^39,95,96^ Two groups received 3–6 μg of the CoronaVac vaccine, and participants aged 3–17 years received 1.5–3 μg. The control group received the same amount of aluminum hydroxide diluent. None of the participants had a history of SARS-CoV-2 exposure or infection, their body temperature was <37 °C, and none was allergic to the vaccine components. The serum neutralizing antibody titer of the subjects was analyzed with a minimum quadruple dilution using microcytosis. The vaccine induced higher titers in children and adolescents groups in the Phase II trial (3 μg adolescent group, 1:142.2; 6 μg adult group, 1:65.4; 6 μg elderly group, 1:49.9). One case of severe pneumonia unrelated to the vaccine was reported in the placebo group in children and adolescents, one case of acute hypersensitivity after the first dose of injection was reported in the adult group, and seven cases of severe adverse reactions were reported in the elderly group. The remaining adverse events were mild or non-toxic. These findings indicated that CoronaVac could be used in children and adolescents, and it is safe for children, adolescents, and adults.

Furthermore, Sinovac performed Phase III (NCT04582344) and IV clinical trials of CoronaVac for patients with autoimmune diseases and rheumatism (NCT04754698).^40,97^ In the Phase III trial, 1413 participants, were analyzed for immunogenicity; 880 of 981 (89.7%) serum samples in the vaccine group were positive for RBD-specific antibodies, compared to 4.4% in the control group. The titer of neutralizing antibodies in 387 sera samples in the vaccine group ranged from 1:15–1:625 (1:15, 16%; 1:75, 38.7%; 1:375, 21%), indicating that most vaccine recipients could produce neutralizing antibodies after vaccination. No deaths or grade IV adverse events occurred in the Phase III trial. In the Phase IV clinical trial, using the above analysis based on microcytosis, the serum neutralizing antibody titer of vaccines with rheumatism was only 1:27 6 weeks after the second dose, which was lower than healthy subjects (1:67). These findings indicated that the dose should be increased for individuals with immune diseases, or the immune adjuvant should be replaced to improve protection. Seven Phase IV clinical trials of the vaccine are in progress (NCT04911790, NCT04953325, NCT04962308, NCT04993365, NCT05107557, NCT05165732, and NCT05148949) (https://clinicaltrials.gov).

According to the study of Chen Y and colleagues,^98^ serum-neutralizing activity against D614G, B.1.1.7(Alpha), and B.1.429 variants after inoculation with CoronaVac were equally effective, while B.1.526, P.1(Gamma) and Beta significantly reduced serum neutralization efficiency. Fernández et al. tested serum neutralization in 44 individuals after two doses of the CoronaVac vaccine. Alpha and Gamma variants could escape from the neutralization of antibodies induced by the vaccine, with escape rates of 31.8 and 59.1% in the subjects, respectively.^99^ Estofolete et al.^100^ reached a similar conclusion that although the CoronaVac vaccine cannot completely inhibit the infection caused by the Gamma variant, the vaccination can help to reduce patients’ clinical symptoms and the rate of death and hospitalization. The Omicron variant can escape neutralizing antibodies elicited by BNT162b2 or CoronaVac, bringing a challenge to existing vaccines.^101^

#### AZD1222

Phase I/II clinical trials of AZD1222 were divided into two stages (NCT04324606).^50,102^ In the first stage, 1077 healthy subjects aged 18–55 years with negative laboratory-confirmed SARS-CoV-2 infection or COVID-19 symptoms were recruited. Ten individuals were injected with two doses of 5 × 10^10^ viral particles (VPs), the remainders were injected with a single dose of 5 × 10^10^ VPs. Those in the placebo group were injected with a licensed meningococcal group A, C, W-135, and Y conjugate vaccine (MenACWY). Serum neutralizing antibody levels were evaluated using a standardized ELISA protocol. The median level of serum samples on day 28 after one dose was 157 ELISA units (EU). The median level of 10 individuals injected with the enhancer dose was 639 EU on day 28 after the second dose, indicating that two injection doses can induce higher neutralizing antibodies. In the second stage of the trial, 52 subjects who had been injected with the first dose received a full-dose (SD) or half-dose (LD) of AZD1222(ChAdOx1 nCoV-19) vaccine on days 28 and 56. The titers of 80% virus inhibition detected by the microneutralization assay (MNA80) were 1:274 (day 0, 28 SD), 1:170 (day 0, 56 LD), and 1:395 (day 0, 56 SD) respectively. The highest titer was produced after the full-second dose injection on day 56. In addition, the AZD1222 vaccine can also induce Th1 biased CD4^+^ and CD8^+^ T-cell responses and further promote cellular immunity. No serious adverse reactions were reported in any phase of the trial, and prophylactic paracetamol treatment reduced the rate of mild or moderate adverse reactions.^103^

In a single-blind, randomized, controlled Phase II/III trial of AZD1222 (NCT04400838),^104^ participants were divided into three groups based on age: 18–55, 56–69, and >70 years. The 18–55 years old group was allocated two low doses (2.2 × 10^10^ VPs)/two standard doses (3.5–6.5 × 10^10^ VPs) ChAdOx1 nCoV-19 and placebo at 1:1 and 5:1, respectively. The 56–69-year-old group was injected with a single dose of ChAdOx1 nCoV-19, a single dose of placebo, two doses of ChAdOx1 nCoV-19, and two doses of placebo (3:1:3:1, respectively). The >70-year-old group was administered a single dose of ChAdOx1 nCoV-19, a single dose of placebo, two doses of ChAdOx1 nCoV-19, and two doses of placebo (5:1:5:1, respectively). All placebo groups received the aforementioned MenACWY vaccine. MNA80 was used to evaluate the titer of serum neutralizing antibodies. The titer of the low-dose group ranged from 1:143 to 1:161, and that of the standard-dose group ranged from 1:144 to 1:193, indicating that ChAdOx1 nCoV-19 can induce high-level neutralizing antibody in all age groups and that two doses of injection can produce higher antibody levels. Thirteen serious adverse events were reported as of October 26, 2020, and none related to vaccine injection. Phase IV clinical trials of the vaccine are in progress (NCT04760132, NCT04914832, NCT05057897, and NCT05142488) (https://clinicaltrials.gov).

Supasa et al. tested the neutralizing effect of AZD1222 on the Alpha variant. GMTs of serum neutralizing antibody decreased by 2.5 times on day 14 and 2.1 times on day 28 after the second dose, while no immune escape was observed.^105^ Subsequently, the neutralization effect of AZD1222 on the Beta variant was tested. On day 14 or 28 after the second dose, the GMTs of the subjects’ serum neutralizing antibodies against the Beta variant were approximately nine times lower than that of the Victoria variant (an early Wuhan-related viral isolate).^106^ In addition, the serum neutralizing antibody GMTs of AZD1222 subjects against the Delta variant decreased by ~4 times compared with the wild type.^107^ On the 28th day after the booster dose, the neutralization ability against Omicron was reduced by about 12.7-fold compared with Victoria and 3.6-fold with B.1.617.2 (Delta).^108^ These findings indicate that the Omicron and Beta variants have stronger immune escape ability than the Alpha and Delta variants. Monitoring vaccine neutralization ability should be highlighted, and existing vaccines should be optimized or strengthened to maintain vaccine efficacy for emerging SARS-CoV-2 variants.

#### Ad26.COV-2-S

Janssen performed Phase I and Phase I-II clinical trials of Ad26.COV-2-S (NCT04436276).^29,30^ A total of 25 healthy adults aged 18–55 with negative nasopharyngeal PCR and serum IgG results participated in the Phase I trial. The participants were equally allocated to receive two doses of low-dose (5 × 10^10^ VPs) Ad26.COV-2-S (low-dose/low-dose, LL), one dose of low-dose vaccine and one dose of placebo (low-dose/placebo, LP), two doses of high-dose (1 × 10^11^ VPs) (high-dose/high-dose, HH), one dose of high-dose vaccine and one dose of placebo (high-dose/placebo, HP), or two doses of placebo (placebo/placebo, PP). The placebo group received a 0.9% sodium chloride solution. The GMTs of serum neutralizing antibody based on the inhibition of 50% of pseudovirus (ID_50_) were detected 14 days after the second dose. The ID_50_ values were 1:242 (LL), 1:375 (LP), 1:449 (HH), and 1:387 (HP) in the vaccine groups. Moreover, Ad26.COV-2-S induced CD4^+^ and CD8^+^ T-cell responses, simultaneously inducing cellular immunity. Adverse events after vaccination were not evaluated in this study.

In the Phase I-IIa clinical trial, 805 healthy adults aged 18–55 and >65 years were equally divided into LL, LP, HH, HP, and PP groups (low-dose: 5 × 10^10^ VPs, high-dose: 1 × 10^11^ VPs). On day 71 or 72 (2 weeks after the injection of the second dose), serum neutralizing antibody GMT based on 50% virus inhibition (IC_50_) of the 18–55-year-old group was 1:827 (LL, day 72), 1:1266 (HH, day 72), 1:321 (LP, day 71), and 1:388 (HP, day 71). On day 29, the serum GMT of the participants injected with a single dose of low-dose or high-dose vaccine in the >65-year-old group was 1:277 or 1:212, respectively. These findings indicated that two injection doses significantly improved antibody titers and enhanced protection. On day 15, 76–83% of the participants in the 18–55 age group and 60–67% of participants in the >65 age group had a Th1 biased CD4^+^ T-cell response, consistent with the results observed in the Phase I trial. After the first dose, most of the reported local adverse events were grade 1 or 2. The most common event was injection site pain. These collective findings indicated that Ad26.COV-2-S is safe. Four Phase IV clinical trials of the vaccine are ongoing (EUCTR2021-002327-38-NL, NCT05030974, NCT05037266, and NCT05075538) (https://www.ncbi.nlm.nih.gov, https://clinicaltrials.gov).

Alter et al. systematically evaluated the neutralization efficacy of the Ad26.COV-2-S vaccine against SARS-CoV-2 variants.^109^ Pseudovirus neutralization test results showed the neutralization titer of the antibody induced by the Ad26.COV-2-S to Gamma variant was 3.3 times lower than the wild type. The neutralization of the Beta variant was five times lower than that of the wild type. The live virus neutralization test showed that the neutralization activity of this variant (Beta) dropped approximately ten times in titers. Garcia Beltran et al. found the neutralization activity of serum samples from Ad26. COV-2 vaccinees against the Omicron variant was reduced by 17 times.^110^

#### NVX-CoV2373

NVX-CoV2373 is a protein subunit vaccine based on the full-length S protein of pre-fusion conformation (rSARS-CoV-2). Relevant Phase I-II clinical trial (NCT04368988) data has been released.^31^ A total of 131 healthy men and non-pregnant women aged 18–59 years were enrolled. All participants had no history of COVID-19 infection and had a low risk of COVID-19 exposure. Among them, six participants were assigned 5 μg/25 μg rSARS-CoV-2 + Matrix-M1 at a ratio of 1:1 as an initial safety measure and were observed for 48 h. The remaining 125 participants received 9% saline (placebo) as group A, two doses of 25 μg rSARS-CoV-2 without adjuvant Matrix-M1 as group B, two doses of 5 μg rSARS-CoV-2 + 50 μg Matrix-M1 as group C, two doses of 25 μg rSARS-CoV-2 + 50 μg Matrix-M1 as group D, and one dose of 25 μg rSARS-CoV-2 + 50 μg Matrix-M1 as group E, at a ratio of 1:1:1:1:1, respectively. ELISA-based neutralization test was used to detect the antibody titers on the 14th day after the second dose. Group C and D showed the most efficacy with the titers of 1:3906 and 1:3305, respectively, four to six times more than convalescent serum. In addition, T-cell responses were also induced and boosted by the adjuvant Matrix-M1. No serious adverse event was reported in this trial except a subject terminated the second dose due to mild cellulitis.

Results of the Phase III clinical trial of NVX-CoV2373 have also been released.^111^ This trial included 16,645 healthy men, non-pregnant women, and people with chronic diseases aged 18–84 without COVID-19 infection and immune disease history. The recipients received two doses of 5 μg NVX-CoV2373 or equivalent placebo (0.9% saline) at a ratio of 1:1. The rate of COVID-19 or SARS-CoV-2 infection 7 days after the vaccination was ~6.53 per thousand in the vaccine group versus 63.43 per thousand in the control group, indicating an overall efficacy of 89.7%. Based on the analysis of subgroups, the effectivity of NVX-CoV2373 in people aged over 65 was 88.9%, and the efficacy against the Alpha variant was 86.3%. The overall rate of adverse events among the recipients was higher in the vaccine group than in the placebo group (25.3 vs. 20.5%). The proportion of serious adverse events was similar in both groups, at about 1%, with one person in the vaccine group reporting severe myocarditis. The vaccine and placebo groups reported one death caused by respiratory failure and one sepsis caused by COVID-19 infection.

A clinical trial was further performed to evaluate the efficacy of NVX-CoV2373 in AIDS patients, in which the Beta variant infected most people. The results indicated that this vaccine showed 60.1% efficacy in HIV-negative participants, indicating that the NVX-CoV2373 vaccine was efficacious in preventing COVID-19.^112^

#### mRNA-1273

Similar to the viral vector vaccines, mRNA vaccines, especially mRNA-1273, also induced Th1 biased CD4^+^ T-cell responses in clinical trials.^28,113^ Moderna performed a Phase I clinical trial of mRNA-1273 (NCT04283461). In the first stage, 45 healthy adults aged 18–55 received two doses of 25, 100, and 250 μg mRNA-1273 at a ratio of 1:1:1. In the second stage, 40 subjects aged >56 years were injected with two doses of 25 and 100 μg vaccine at a ratio of 1:1. The interval between all injections was 28 days. There was no control group. PRNT_50_ was used to detect the titers of serum neutralizing antibodies in different age groups 14 days after the second dose, and the titers were 1:343.8 (100 μg, 18–55 years old), 1:878 (100 μg, 56–70 years old), and 1:317 (100 μg, >70 years old). The vaccine induced potent neutralizing antibodies in different age groups, and the highest titer was induced in the 56–70 age group. After the first dose, 23 participants aged 18–55 (51.1%) reported systemic adverse reactions. All the adverse reactions were mild or moderate. After the second dose, three subjects reported serious adverse reactions. No serious adverse events occurred in the group aged over 56 years.

Moderna also performed a Phase III clinical trial of the mRNA-1273 vaccine. The number of participants was 30,420, aged over 18 years and had no history of SARS-CoV-2 infection. Subjects were injected with two doses of mRNA-1273 vaccine (100 μg) at a 28-day interval or with normal saline at a 1:1.^114^ From the first day to November 25, 2020, 196 cases of COVID-19 were diagnosed by preliminary analysis, with 11 cases in the vaccine group and 185 cases in the placebo group, indicating a 94.1% effectiveness of mRNA-1273. After the first dose, adverse events occurred in 84.2% of the participants in the vaccine group, and 88.6% of the participants in the vaccine group reported adverse events after the second dose. The adverse events were mainly graded 1 or 2.

Furthermore, there were three deaths in the placebo group (one each from intraperitoneal perforation, cardiopulmonary arrest, and systemic inflammatory syndrome) and two deaths in the vaccine group (one from cardiopulmonary arrest and suicide). Although the death rate was low and unrelated to vaccination, the effects of nucleic acid vaccines on cardiopulmonary and other functions still need to be further studied. Phase IV clinical trials of the mRNA-1273 vaccine are currently underway (NCT04760132, NCT05060991, NCT04952402, NCT05030974, NCT05047718, NCT05075538, and NCT05075538) (https://clinicaltrials.gov).

The mRNA-1273 vaccine is still effective for the Alpha variant, but its neutralization effect on the Beta variant is reduced. The pseudovirus neutralization test showed that the antibody titers of mRNA-1273 against the Beta variant were 6.4 times lower than that of the D614G mutant.^115^ McCallum et al. tested the neutralization efficacy of mRNA-1273 against the B.1.427/B.1.429 variant and found that the neutralizing antibody GMTs induced by the vaccine decreased by 2–3.5 times compared to the wild type.^116^ Furthermore, more than 50% of mRNA-1273 recipients’ serum failed to neutralize the Omicron variant, with the GMTs reduced by about 43 times.^110,117^

#### BNT162b2

Phase I and III clinical trials of the BNT162b2 mRNA vaccine have also been performed (NCT04368728).^117^ The Phase I clinical trial performed by Pfizer-BioNTech involved two candidate vaccines, BNT162b1 encoding RBD and BNT162b2 encoding the full-length of S protein. This trial included 185 healthy adults aged 18-55 and 65–85. With 15 individuals per group, they were divided into 13 groups (seven groups aged 18–55 and six groups aged 65–85) and inoculated with two doses of 10/20/30 μg BNT162b1 or BNT162b2, and an additional group aged 18–55 received a single dose of 100 μg BNT162b2. Twelve individuals in each group were vaccinated with BNT162b1/BNT162b2, and three were vaccinated with a placebo. The 50% neutralization titers were determined on the 14th day after the second dose, ranging from 1:33 to 1:437 (BNT162b1) and 1:81 to 1:292 (BNT162b2). BNT162b1 and BNT162b2 both induced high-level production of antibodies. The local adverse reactions caused by these two vaccines were similar, mainly pained at the injection site. However, the overall rate of adverse events of BNT162b2 was low, with less use of antipyretic analgesics and these findings indicated that BNT162b2 is safer.

The Phase III clinical trial involved 43,548 participants aged 16 years and over, who were injected with two doses of BNT162b2 (30 μg at an interval of 21 days) or placebo at a ratio of ~1:1.^118^ At least 7 days after the second dose, eight cases of COVID-19 were observed in the vaccine group, while 162 cases of COVID-19 were observed in the placebo group, indicating the effectiveness of 94.6%. Mild-to-moderate pain at the injection site within 7 days of the first dose of BNT162b2 was the most common local adverse reaction. Less than 1% of all subjects reported severe pain, and none of the participants reported grade 4 local adverse reactions. Two BNT162b2 vaccinees died (one from arteriosclerosis and one from cardiac arrest), four placebo subjects died (two from unknown causes, one from hemorrhagic stroke, and one from myocardial infarction). None of the deaths was related to the vaccine or placebo. Like the mRNA-1273 vaccine, heart disease also occurred in the BNT162b2 vaccine injection group, indicating that the mRNA vaccine needs to be strictly evaluated. Phase IV clinical trials of the BNT162b2 vaccine are currently underway (NCT04760132, NCT05060991, NCT04961229, NCT04775069, NCT04878211, NCT04952766, NCT04969250, NCT05047718, NCT05057169, NCT05057182, and NCT05075538) (https://clinicaltrials.gov).

Collier et al. tested the neutralization efficacy of the sera of single-dose BNT162b2 vaccine subjects against the Alpha variant.^119^ Ten of 23 samples showed a decrease in neutralization efficacy, with a maximum decrease of about six times. Supasa et al. showed that the neutralization activity of the BNT162b2 vaccine against the Alpha variant decreased by 3.3 times.^105^ Subsequently, the researchers further tested the neutralization activity of BNT162b2 against the Beta variant and found that the GMTs of neutralizing antibodies decreased by 7.6 times.^106^ In addition, the neutralization activity of the BNT162b2 vaccine against Kappa, Delta, B.1.427, and B.1.429 variants was reduced by at least two times (Kappa and Delta), 1.2 times (B.1.427), and 1.31 times (B.1.429).^120^ Although the Delta variant has high infectivity and can cause immune escape, Liu et al. reported that BNT162b2 retained neutralizing activity against the delta variant.^121^ In the study carried out by Cameroni E and colleagues, the neutralization activity of BNT162b2 booster-dose recipients’ serum significantly increased, but its neutralization capability against the Omicron variant still decreased by at least fourfold compared with the Wuhan-Hu-1 strain.^122^

### The effectiveness of COVID-19 vaccines in the real world

Although clinical trials can reflect the effectiveness of vaccines, the outcomes are partly dependent on the status of participants. Thus, the data were not very objective. The real-world study can help to establish clinical trial evidence and provide information for adjusting the vaccination strategy. Here, we summarize several current real-world studies to support these vaccines’ efficacy further. A study on the effectiveness of mRNA vaccine in American healthcare workers (HCW) showed that the overall efficacy of BNT162b2 and mRNA-1273 vaccines were 88.8 and 88.9%, respectively.^123^ A study involving six locations in the United States, HCW, and the first responders also showed that after two doses of mRNA vaccine, the effective rate was about 90%.^124^ In addition, the 2nd dose of BNT162b2 was shown to reduce 94% of COVID-19 cases in a 1.2 million person dataset.^125^ A large-scale study in Scotland showed that the first BNT162b2 vaccination could achieve an efficacy of 91%, and the number of COVID-19 hospitalization decreased in 28–34 days after vaccination. The efficacy of AZD1222 in the same period was 88%, and these two vaccines showed a similar effect on preventing infection.^126^ There are limited real-world data on inactivated vaccines. The effectiveness of the CoronaVac vaccine was evaluated in a St. Paul study and showed more than 50% efficacy.^127^

These real-world studies showed that the approved COVID-19 vaccines effectively prevent SARS-CoV-2 infections, especially reducing the infection in susceptible people like healthcare workers.

### Variants of Concern (VOC)

**Fig. 7:**
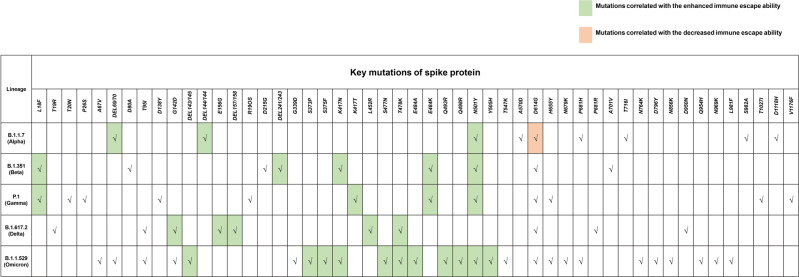
A systemic illustration of the mutation in the S protein of VOCs. VOCs were designated by WHO because of the enhanced infectivity or immune escape ability (or with both), the specific mutations in the S protein of VOC Alpha to Omicron are displayed, and the mutations related to enhanced immune escape ability were marked in green color, while the mutation related to decreased immune escape ability was marked into orange color

As mentioned earlier, the emergence of VOC poses great challenges to the efficacy of existing vaccines. WHO has designated five VOCs, including Alpha, Beta, Gamma, Delta, and Omicron (Fig. 5), among which Alpha and Delta variants had strong contagious activity, while Beta and Gamma variants gained powerful immune escape ability. However, the Omicron variant obtained high infectivity and can evade most COVID-19 vaccines simultaneously. Understanding the relationship between the mutations and pathogenic characteristics (like infectivity and immune escape ability) is useful to analyze the efficacy of vaccines better and adjust the vaccination strategy properly. Here, the origin of these VOCs has been systematically reviewed, and the influence of mutations on the pathogenic characteristics is illustrated (Fig. 7). Furthermore, the effectiveness of approved vaccines on the Omicron variant was also discussed, given that the Omicron variant has caused large-scale infections worldwide and aroused people’s worries.

#### Alpha

B.1.1.7 is the first variant circulating worldwide, which was first detected in the southeast of the UK in September 2020 and became the dominant variant in the UK during the following 3 months. On December 18, 2020, B.1.1.7 was designated as Variants of Concern (VOC) and labeled Alpha by WHO (https://www.who.int/en/activities/tracking-SARS-CoV-2-variants/). Compared with other variants at that time, the Alpha variant had a stronger transmission ability, with a higher reproduction number.^128^ Interestingly, the variant lineage contained three subgroups initially, but the variant with Del69/70 in the S protein eventually occupied the mainstream, and 96.6% of all detected sequences of Alpha variants contained the mutation (https://outbreak.info/), which indicated the existence of selective advantage in the transmission of SARS-CoV-2.^12^ Apart from Del69/70, other mutations (like D614G in each VOC and E484K in Beta and Gamma) also proved the selective advantage. Variants with certain mutations gained stronger infectivity, fitness, or immune escape ability and are prone to survive and spread in the struggle between humans and COVID-19.

The analysis of these mutations with the selective advantage will further help to understand the pathogenic characteristics of these variants, such as infectivity, contagious ability, and immune escape ability. In addition to Del69/70, there are eight mutations in the S protein of Alpha variant: Del144 (contained in 95% of all detected sequences of Alpha variants), N501Y (97.6%), A570D (99.2%), D614G (99.3%), P681H (99%), T716I (98.7%), S982A (98.8%), and D1118H (99.2%) (https://outbreak.info/). Among these mutations, Del69/70 and Del144 can significantly reduce the neutralization of NTD targeted antibodies,^105^ because most of the immune epitopes of NTD antibodies are located in N3 (residues 141-156) and N5 (residues 246–260) loops, while Del144 can alter the N3 loop and cause the immune escape of such antibodies,^129^ Del69/70 can enhance the infectivity.^130^ The characteristic mutation N501Y can significantly increase the binding of S protein to ACE2,^131^ and further enhance the infectivity. In addition, N501Y was also related to the immune escape, in which the epitope of class A antibodies was located.^129^ This mutation was also in other VOCs like Beta, Gamma, and Omicron. Not only VOC, but almost all circulating variants also had a D614G mutation. Plante JA et al. found that D614G can alter the fitness and enhance the replication of SARS-CoV-2 in the lungs. However, D614G will reduce the immune escape ability of the virus and improve the sensitivity to neutralizing antibodies.^131,132^ The above studies suggested that this mutation may be essential to maintaining the survival of SARS-CoV-2. Thereby, it can be retained continuously. The P681H mutation near the furin-cleavage site may enhance the cleavage of S1 and S2 subunits and increase the Alpha variant’s entry. The P681R in VOC Delta may improve fitness compared with P681H in the Alpha variant.^133^

In general, the Del69/70, N501Y, D614G, and P681H of the Alpha variant were helpful to improve the infection, which can explain the high reproduction number of about 3.5–5.2 (https://aci.health.nsw.gov.au/covid-19/critical-intelligence-unit/sars-cov-2-variants). However, Del144 and N501Y affected the neutralization of antibodies, the vaccines approved by WHO showed strong neutralization ability to VOC Alpha, shown in Table 3.

**Table 3:** Efficacy of COVID-19 vaccine against SARS-CoV-2 VOCs

SARS-CoV-2 variant of concern (VOC)	Transmissibility (compared with wild type)*	Virulence and reinfection*	Efficacy of COVID-19 vaccine						
			BBIBP-CorV	CoronaVac	AZD1222	Ad26.COV-2-S	NVX-CoV2373	mRNA-1273	BNT162b2
B.1.1.7 (Alpha)	3.5–5.2	Increased risk of hospitalization, possible increased risk of mortality. Increased secondary attack rate.	Unknown	Unknown	70.4%^261^	70.2%^262^	85.6%^262^	1.2-fold decrease of neutralizing antibody titers^263^	89.5%^264^
B.1.351 (Beta)	5.2	Possible increased risk of severe disease and in-hospital mortality.	Two-dose: 5.47-fold decrease of plasma neutralization titers compared to against prototype. Homologous booster: 10.02-fold increase. BBIBP-CorV/ZF2001 heterologous booster: 32.16-fold increase.^93^	Unknown	83%^265^	51.9%^262^	60%^266^	6.4-fold decrease of neutralizing antibody titers^263^	75%^264^
P.1 (Gamma)	Increasing	Possible increased hospitalization and severe disease. Increased secondary attack rate.	Unknown	50%^267^	48%^268^	36.5%^262^	Unknown	3.5-fold decrease of neutralizing antibody titers^263^	88%^268^
B.1.617.2 (Delta)	3.2–8	Increased risk of emergency care and hospitalization. Increased secondary attack rate.	Two-dose: 1.47-fold decrease of plasma neutralization titers compared to against prototype. Homologous booster: 10.07-fold increase. BBIBP- CorV/ZF2001 heterologous booster: 17.65-fold increase.^93^	Showed good results.^267^	67.0%.^269^	Ad26.COV-2-S /mRNA-1273 heterologous booster: 16-fold increase.^110^	Under investigation	Two-dose: 95%^268^ Homologous booster:threefold increase.^110^	Two-dose: 87–95%^268^ Homologous booster:ninefold increase.^110^
B.1.1.529 (Omicron)	2.6–4.0	Decreases risk of hospitalization and death compared to Delta. Increased secondary attack rate.	Two-dose: 1.21-fold decrease of plasma neutralization titers compared to against prototype. Homologous booster: 9.54-fold increase. BBIBP- CorV/ZF2001 heterologous booster: 5.86-fold increase.^93^	Two-dose: no effect.^231^ CoronaVac/BNT162b2 heterologous booster: 1.4-fold increase compared to two-dose mRNA vaccine.^231^	Two-dose: no effect. AZD1222/ BNT162b2 heterologous booster: 62.4% at 2–4 weeks, 39.6% at 10 or more weeks.^270^	Two-dose in previously infected vaccinator: GMNT decrease of 17-fold than wild type. Ad26.COV-2-S /mRNA-1273 heterologous booster: fourfold increase.^110^	Under investigation	Two-dose: GMNT decrease of 43-fold than wild type.^110^ Homologous booster: 70.1% at 2 to 4 weeks, 60.9% at 6–9 weeks.^270^	Two-dose: 30–37%^271^ Homologous booster: 67 .2% at 2 to 4 weeks, 45.7% at 10 or more weeks. BNT162b2/mRNA-1273 heterologous booster: 73.9% at 2–4 weeks, 64.4% at 6–9 weeks.^270^

^*^ https://aci.health.nsw.gov.au/covid-19/critical-intelligence-unit/sars-cov-2-variants

#### Beta

B.1.351 (also known as 501Y.V2) was first detected in South Africa in May 2020 and firstly appeared after the first epidemic wave in Nelson Mandela Bay. This variant had different characteristics from the dominant variants B.1.154, B.1.1.56, and C.1 in the first wave of pandemic^134^ and had spread rapidly in Eastern Cape, Western Cape, and KwaZulu-Natal provinces in just a few weeks, causing the second wave of epidemic in South Africa (October 2020).^135^ On December 18, 2020, B.1.351 was designated as VOC by WHO and named Beta (https://www.who.int/en/activities/tracking-SARS-CoV-2-variants/). Similar to the Alpha variant, B.1.351 lineage also included three subtypes 501Y.V2-1/-2/-3, and 501.Y.V2-1 occupied mainstream, then the 501Y.V2-2 with additional mutations of amino acid site 18 and 417 appeared, and finally Del241/243 mutation occurred in 501Y.V2-3.^136^ Among all detected sequences of VOC Beta, 89.6 and 93% had K417N and Del241/243 mutations, indicating that 501Y.V2-3 was the dominant subgroup of VOC Beta (https://outbreak.info/).

There were nine mutations in the S protein of Beta variant: L18F (found in 43.6% reported Beta variants), D80A (97.1%), D215G (94.6%), Del241/243 (89.6%), K417N (93%), E484K (86.5%), N501Y (87%), D614G (97.8%), and A701V (96.4%) (https://outbreak.info). The glycans of amino acid site 17, 174, 122, and 149 in the NTD region combined into seven targeted epitopes of NTD antibodies^137^ and L18F may interfere with the binding between antibodies, and residue 17 affect the neutralization of antibody. The Del241/243 map to the same surface as the Del144 in the Alpha variant,^138^ which may also interfere with the neutralization of antibodies. In addition, several studies have shown that K717N and E484K mutations (as well as the K417T in Gamma variant and E484A in Omicron variant) both contribute to the immune escape against group A-D antibodies,^129,136,139,140^ and K417N can enhance the infectivity at the same time.^129,141^

Overall, the L18F, Del241/243, K417N, E484K, and N501Y mutations all contribute to the immune escape ability of VOC Beta, while K417N, N501Y, and D614G can enhance the viral infection. Therefore, compared with the Alpha variant, the Beta variant has poor transmissibility, but a very strong immune escape ability and can reduce the neutralization efficacy of WHO-approved vaccines by more than 10 times.

#### Gamma

P.1 was first detected in Brazil in November 2020 and caused the second wave of the epidemic in this country, causing more than 76% infection of the population,^142^ and the average number of daily-confirmed COVID-19 patients in Manaus increased by 180 from January 1 to 19, which was about 30 times of the average increased cases in December. On January 11, 2021, P.1 was designated as VOC by WHO and labeled Gamma.

There were 12 mutations in the S protein of Gamma variant: L18F (found in 97.9% reported P.1 strains), T20N (97.9%), P26S (97.6%), D138Y (95.5%), R190S (93.6%), K417T (95.5%), E484K (95.2%), N501Y (95.3%), D614G (99%), H655Y (98.5%), T1027I (97.2%), V1176F (98.1%) (https://outbreak.info). Since most of the mutations of interest like K417T, E484K, N501Y, and D614G have been introduced in the Alpha and Beta variants mentioned above, they will not be repeated here.

Among these mutations, L18F, K417T, E484K, and N501Y help to enhance the immune escape ability, while K417T, N501Y, and D614G can enhance the viral infection. Therefore, VOC Gamma showed a similar immune escape ability to VOC Beta, but less than the Beta variant, which may be caused by mutations outside the RBD region,^143^ the infectivity of both Beta and Gamma variants were less than the Alpha variant (https://aci.health.nsw.gov.au/covid-19/critical-intelligence-unit/sars-cov-2-variants).

#### Delta

B.1.617.2 was first detected in Maharashtra, India, in October 2020 and spread rapidly in a few months due to the relaxation of prevention and control measures for COVID-19, causing the death of more than 400,000 people.^107^ On May 11, 2021, this variant was designated as VOC by WHO and labeled Delta (https://www.who.int/en/activities/tracking-SARS-CoV-2-variants/). VOC Delta was a worldwide circulating VOC after VOC Alpha and was detected by at least 169 countries (https://outbreak.info).

There were ten mutations in the S protein of Delta variant: T19R (found in 98.3% reported delta strains), T95I (38.3%), G142D (66.1%), E156G (92.1%), Del157/158 (92.2%), L452R (96.9%), T478K (97.2%), D614G (99.3%), P681R (99.2%), D950N (95.3%) (https://outbreak.info). G142D and E156G are located in the N3 loop, which NTD antibodies could target,^129^ thus may affect the neutralization activity of NTD antibodies. The Del157/158 map to the same surface as the Del144 in the Alpha variant and the Del241/243 in the Beta variant, respectively, which may affect the neutralization of antibodies.^138^ In addition, both L452R and T478K are located in immune epitopes targeted by group A-B antibodies, enhancing the immune escape ability of Delta variant,^129,138,144^ and L452R is related to a higher infectivity.^145^ The P681R mutation enhanced the infectivity of the virus and further improved the fitness compared with P681H,^138^ which explained the higher infectivity of VOC Delta than VOC Alpha.

Although the mutations like L452R, T478K have not been reported in previous VOC Alpha, Beta, and Gamma, these mutations gave VOC Delta a stronger transmission ability (with a reproduction number of 3.2–8, mean of 5) and immune escape ability than VOC Alpha, which made Delta variant quickly become a dominant variant and reduce the efficacy of approved vaccines (https://aci.health.nsw.gov.au/covid-19/critical-intelligence-unit/sars-cov-2-variants).

#### Omicron

In November 2021, B.1.1.529 appeared in many countries. Since the S protein of this variant contains more than 30 mutation sites, and many of them coincide with the S protein mutations of previous VOCs, B.1.1.529 was designated as VOC by WHO on 26 November 2021 and labeled Omicron (https://www.who.int/en/activities/tracking-SARS-CoV-2-variants/). Although the Omicron variant has more mutations, the severity of the Omicron infected patient was less than Delta. After infection with the Omicron variant, hamsters did not have progressive weight loss similar to that after infection with Alpha/Beta/Delta, and the number of virus copies in the lungs was lower,^146^ indicating that Omicron has less effect on the lower respiratory tract. By evaluating Omicron infection on different cells, Thomas P. Peacock et al. found that the infection degree of Omicron on Calu-3 (a lung cell line, whoseTMPRSS2 expression is normal, but lack of CTSL expression, hindering the nuclear endosome pathway of virus entry) is weaker than Delta, indicating that Omicron entry is more dependent on the nuclear endosome mediated endocytosis pathway^147^ rather than the membrane fusion pathway involved in TMPRSS2, and TMPRSS2 is mainly distributed in human lung epithelial cells. Therefore, Omicron has less infectivity to the lungs and causes mild symptoms, mainly causing upper respiratory tract infection.

The S protein of the Omicron variant contains 31 mutations: A67V, Del69/70, T95I, G142D, Del143/145, N211I, Del212-212, G339D, S371L, S373P, S375F, K417N, S477N, T478K, E484A, Q493R, G496S, Q498R, N501Y, Y505H, T547K, D614G, H655Y, N679K, P681H, N764K, D796Y, N856K, Q954H, N969K, and L981F (since the proportion of mutations is constantly changing, it is not shown here) (https://outbreak.info). Cao Y and colleagues systematically analyzed the effect of these mutations on immune escape. Among them, 477/493/496/498/501/505 mutations affected the neutralization activity of group A antibodies, 477/478/484 mutations affected the neutralization activity of group B antibodies, while the neutralizing activity of group C/D/E antibodies was affected by 484, 440/446, and 346/440 mutations, respectively, Group F antibodies are disturbed by 373/375 mutations.^94,129^ However, group E and F antibodies showed effective neutralization of the Omicron variant among these antibodies. These two groups of antibodies were rarely used in the clinic and formed lower immune pressure on the virus, reducing the viral mutation of these antibodies and maintaining the binding of antibodies to corresponding epitopes.

Although the Del69/70, K417N, N501Y, D614G, and P681H mutations can enhance the viral infection (with a reproduction number of 2.6–4.0) and Del143/145, K417N, T478K, E484A, and N501Y are related to the immune escape, the infection of Omicron variant has less impact on the lung and is unlikely to cause serious diseases compared with VOC Delta. In addition, many vaccines serum almost lost the neutralization effect on the Omicron variant, indicating that new strategies (such as booster vaccination, sequential vaccination, and the development of new platforms such as nanoparticle vaccine) should be considered.

Pajon et al. and Nemet et al. evaluated the enhanced protection of the third dose of mRNA-1273 and BNT162b2 against the Omicron variant, respectively.^148,149^ Although a booster dose can enhance the response of memory cells and increase the antibody titers to produce stronger neutralization activity of 20 to100-fold, the enhanced immune response is still limited. An Israeli study showed that the fourth dose of the BNT162b2 or mRNA-1273 vaccine still could not prevent Omicron infection (https://www.shebaonline.org/). In addition, Wang J and colleagues evaluated the protection of the fourth BBIBP-CorV against the Omicron variant. Although the additional inoculation successfully recalled memory cell response in the 6th month after the third dose, the production of antibodies targeting the RBD region was suppressed due to the enhanced immune pressure and decreased peak level^150^ The suppression of RBD-targeted antibodies may induce the change of immune epitopes, and a vaccine inducing diverse epitopes antibodies (like a polyvalent vaccine) may decrease the immune pressure on certain epitopes and maintain the efficacy on different VOCs.

SCTV01E is a protein subunit vaccine under development that uses the S trimer of Alpha/ Beta/ Delta/ Omicron variants, and two clinical trials evaluating the safety and immunogenicity of SCTV01E are on the way (NCT05239806 and NCT05238441) (https://clinicaltrials.gov). In addition to the polyvalent vaccine, the mRNA vaccine used the VOC Beta sequence also showed better protection against Omicron in the hamster model than existing vaccines.^151^

### Relevant data of COVID-19 vaccines not yet approved by the WHO

According to the WHO data, as of March 28, 2022, 196 candidate vaccines are in the preclinical stage, and 153 candidate vaccines based on different vaccine platforms have been approved for clinical trials. Here, we present some data for each type of vaccine that the WHO has not approved.

#### Inactivated vaccines

As of March 28, 2022, 12 inactivated virus vaccines underwent Phase II/III and Phase IV clinical trials. Of these Phase III clinical trials, the QazCovid-in^®^-COVID-19 inactivated vaccine developed by the Research Institute for Biological Safety Problems, Republic of Kazakhstan, showed superiority in many aspects, including good immunogenicity and high seroconversion (https://clinicaltrials.gov/ct2/show/NCT04691908).

#### Live attenuated vaccine

As of March 28, 2022, only one live attenuated vaccine-COVI-VAC has entered a Phase III clinical trial (ISRCTN15779782). The vaccine was developed by the Codagenix and Serum Institute of India. The study starts in August 2021 and runs until September 2023 to objectively evaluate the benefit and risk of COVI-VAC as a candidate vaccine, and relevant data have not been released (https://www.isrctn.com/ISRCTN15779782).

#### Viral vector vaccine

As of March 28, 2022, two replicating viral vector platform vaccines and eight non-replicating viral vector platform vaccines have been tested in Phase II/III and Phase IV clinical trials. The Gam-COVID-Vac aroused many concerns owing to its effectiveness of 91.6%.^152^ A Phase III trial was conducted in Moscow on September 7, 2020 (NCT04530396). 21,977 adults were randomly assigned to the vaccine and placebo groups in this trial. The vaccine group received 0.5 mL Gam-COVID-Vac. Only 0.1% of recipients were infected with SARS-CoV-2, while the percentage of the placebo group was 1.3%. No severe adverse events related to the vaccine were reported.

#### Protein subunit vaccine

As of March 28, 2022, 22 candidate protein subunit vaccines were in Phase II/III and Phase IV clinical trials. The CpG 1018/Alum-adjuvanted SCB-2019 vaccine was developed by Clover Biopharmaceuticals Inc. and Dynavax. A Phase III clinical trial (NCT05012787), beginning on August 19, 2021, was conducted to evaluate the safety and immunogenicity of the investigational SCB-2019 in adult participants with stable chronic inflammatory immune-mediated diseases (IMDs) (https://clinicaltrials.gov/ct2/show/NCT05012787). Moreover, an RBD-based subunit vaccine developed by the West China Hospital, Sichuan University, and WestVac Biopharma Co., Ltd, showed strong induction of potent functional antibodies, as well as CD4^+^ T-cell responses in the preclinical trial,^60^ and the phase III clinical trial (NCT04887207) of this vaccine, has been completed (https://clinicaltrials.gov/ct2/show/results/NCT04887207).

#### DNA and mRNA vaccines

As of March 28, 2022, nine RNA and four DNA vaccines have undergone Phase II/III and Phase IV clinical trials. An mRNA vaccine called mRNA ARCoV, developed by the Academy of Military Science, Walvax Biotechnology, and Suzhou Abogen Biosciences was conducted for a Phase III clinical trial of 28,000 subjects (NCT04847102). The subjects were inoculated with a vaccine or placebo in a 1:1 ratio with an interval of 28 days between two injections. It was reported that expected efficacy and good safety had been achieved. The effects of cross-injection will be assessed, including an immunogenic subgroup and a reactive subgroup, to evaluate the humoral immunity induced by the vaccine (https://clinicaltrials.gov/ct2/show/NCT04847102).

## Safety of vaccines

### Vaccine-induced complications

**Fig. 8:**
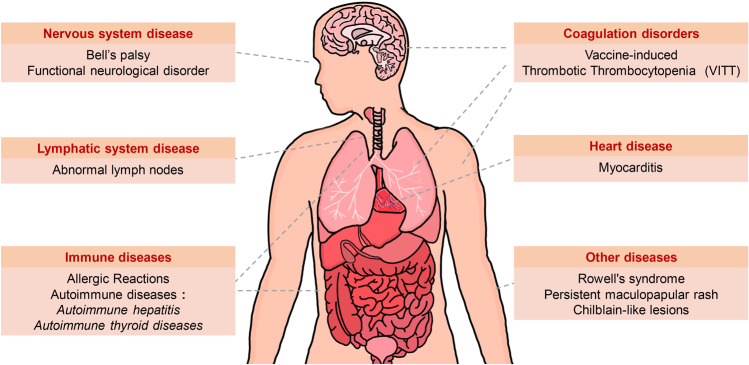
A summary of some possible complications induced by COVID-19 vaccines. The possible complications induced by COVID-19 vaccines mainly include the following categories: (1) coagulation dysfunction, such as thrombocytopenia; (2) heart diseases, such as myocarditis; (3) immune diseases, such as allergic reactions, autoimmune hepatitis, and autoimmune thyroid diseases; (4) nervous system diseases, such as facial paralysis and functional neurological disorders; (5) lymphatic system diseases; and (6) other diseases, such as Rowell’s syndrome, macular rash, and chilblain-like lesions

Although the currently approved COVID-19 vaccines were safe in clinical trials, the resulting adverse reactions are numerous, including fever, headache, fatigue, injection site pain, and nausea.^3,153^ As the vaccination campaign progressed, complications occurred in some subjects, and several patients died of cardiovascular diseases, such as arteriosclerosis. Furthermore, cardiac arrest occurred in Phase III clinical trials of the mRNA-1273 and BNT162b2 vaccines.^114,118^ The possible complications induced by COVID-19 vaccines mainly include the following categories: (1) coagulation dysfunction, such as thrombocytopenia;^52,154^ (2) heart diseases, such as myocarditis;^74,75^ (3) immune diseases, such as allergic reactions,^155^ autoimmune hepatitis,^156^ and autoimmune thyroid diseases;^157^ (4) nervous system diseases, such as facial paralysis^158,159^ and functional neurological disorders;^153^ (5) lymphatic system diseases;^160^ and (6) other diseases, such as Rowell’s syndrome,^161^ macular rash,^162^ and chilblain-like lesions^163^ (Fig. 8). Although the incidence of these complications is low, the relationship between vaccines and these diseases needs to be explored. Here, we describe related COVID-19 vaccine complications and analyze the factors.

#### Blood coagulation dysfunction

Greinacher et al. and Lee et al. reported thrombocytopenia in an adenovirus vector vaccine and mRNA vaccine recipients.^154,164^ A large number of platelet factor 4 (PF4) antibodies were presented in the patients, and the antibody heparin PF4 complex acted on platelet FC γ receptors, activating platelets and further producing procoagulant substances.^154^ Adenoviruses can bind to platelets and activate them.^165,166^ However, trace adenoviruses in vaccines injected one or two weeks before onset seem unlikely to cause platelet activation. Further analysis of PF4 structure revealed that PF4 antibodies from vaccine-induced immune thrombocytopenia patients induced heparin-induced thrombocytopenia by binding eight surface amino acids on PF4.^51^ One study counted the cases of thrombosis sequelae voluntarily reported after vaccination, of which at least 169 cases of possible cerebral venous thrombosis and 53 cases of possible visceral venous thrombosis were reported among 34 million individuals vaccinated with ChAdOx1 nCoV-19 vaccine, and 35 cases of central nervous system thrombosis among 54 million individuals vaccinated with BioNTech mRNA vaccine. Among the 4 million subjects receiving the Moderna mRNA vaccine, cerebral venous sinus thrombosis may have developed in five cases. Among the more than 7 million subjects receiving Ad26.COV-2-S vaccine, cerebral venous thrombosis may have developed in six cases.^52^ Although the relevant pathogenesis is unclear, a possible trigger factor for these PF4 antibodies is free RNA or DNA in the vaccine.^167^

Moreover, platelet activation may also relate to the injury and inflammation induced by mast cell (MC) degranulation. Wu ML et al. found that SARS-CoV-2 can induce degranulation of MCs located in the mucosa, and a rapid MC degranulation could be recapitulated through the binding of RBD to ACE2, resulting in supra-alveolar dermatitis and lung injury.^168^ In addition, in the case of inflammation induction and lung epithelial injury, many plasminogen activators may be released.^169^ Thus, the increased D-dimer (one of the products formed when plasminase degrades fibrine) concentration was observed in many COVID-19 patients, with a decreased level of platelets.^169^ These pathological characteristics of patients were very similar to the thrombotic thrombocytopenia caused by the COVID-19 vaccination. Combined with the above studies, this mechanism may be explained as follows: after the SARS-CoV-2 infection or mRNA vaccine vaccination, S protein stimulated lung epithelial cells and induced MC degranulation, increasing the level of inflammatory mediators. These mediators increased the destructive effect of monocyte macrophages on erythrocytes and led to abnormal platelet levels. In addition, the injury of epithelial cells activated platelets and released coagulation factors, finally forming fibrin and forming extensive micro thrombosis. In this process, the over-consumed platelets and coagulation factors lead to the reduction of coagulation activity, further imbalance of coagulation and anticoagulation, secondary hyperfibrinolysis, and the release of a large number of plasminogen activators, eventually leaded to disseminated intravascular coagulation (DIC), which appeared in most COVID-19 patients.^154,169,170^ Compared with COVID-19 patients, fewer mRNA vaccine subjects reported DIC, which may be due to the lower amount of S protein produced after vaccination than natural infection, and the inflammation is also lower.

Relevant indexes (e.g., measuring prothrombin time, platelet count, and D-dimer concentrations of the receptors) should be tested within 2–3 days after vaccination to prevent the platelet abnormalities caused by COVID-19 vaccination.^169^ For patients with abnormal index, preventive treatment (usually heparin or low molecular weight heparin transfusion, the latter is safer) should be taken as soon as possible.^169^ In addition, degranulation inhibitors may also be a feasible means to inhibit the inflammatory response and prevent lung injury and platelet abnormalities.^168^

#### Heart diseases

Myocarditis is a rare cardiac complication after COVID-19 vaccine injection.^74,75^ Rosner et al. reported seven patients hospitalized for acute cardiomyoid disease after vaccination with Pfizer-BioNTech/AstraZeneca (*n* = 6) and Janssen (*n* = 1) vaccines. Larson et al. reported eight patients hospitalized for chest pain within 2–4 days of vaccination with the BNT162b2 or mRNA-1273 vaccine. The laboratory diagnostic cardiac magnetic resonance imaging analysis revealed that these patients have myocarditis. All the subjects had left ventricular ejection dysfunction. The median ejection blood percentage was 48–59%.^74,75^ These two studies showed a significant temporal correlation between mRNA-based COVID-19 vaccines (including viral vector and mRNA vaccines) and myocarditis. Such systemic adverse events usually occur within 48 h after the second dose.^114,118^ There may be two potential mechanisms for COVID-19 mRNA vaccines causing heart diseases, such as myocarditis. The first is the nonspecific innate inflammatory responses induced by mRNA. The second is the interaction of the S protein produced by mRNA after the translation within the heart or blood vessels, resulting in cardiovascular injury.^171^ Since protein subunit vaccines like ZF2001 and NVX-CoV2373 have not been used widely, and the relevant data are still unreleased, it is not easy to judge whether the S protein causes myocarditis.

#### Immune diseases

Immune diseases caused by the injection of the COVID-19 vaccine mainly include allergic reactions and autoimmune diseases that include autoimmune hepatitis and autoimmune thyroid diseases.^155–157^

##### Allergic reactions

From December 14 to 23, 2020, 175 of the first batch of 1,893,360 individuals vaccinated with BNT162b2 developed severe allergic reactions within 24 h. These cases were submitted to the vaccine adverse events reporting system (VAERS).^155^ Finally, 21 cases were identified as allergic reactions based on the Brighton Collaboration definition criteria.^155,172,173^ Between December 21, 2020, and January 10, 2021, ten of the 4,041,396 subjects vaccinated with the first batch of mRNA-1273 vaccine were identified as allergic reactions.^155,173^ Risma et al. analyzed the causes of allergic reactions induced by the COVID-19 vaccine. The reasons included nucleic acid of COVID-19 vaccine activated contact system; complement system that was directly activated by the nano lipid plasmid (LNP) vector of the vaccine, resulting in complement-related pseudoanaphylaxis;^174^ pre-existing antibodies to polyethylene glycol (PEG) that induced allergic reactions;^175^ and direct activation of mast cells leads to degranulation. Allergic reaction mainly includes classical pathway and non-classical pathway. The classical pathway is activated by mast cells and cross-linked IgE,^176^ which PEG IgE antibodies may activate in the inoculant. Non-classical pathways mainly involve complement antibody-dependent activation of mast cell activation.^177^ To further understand the causes of allergic reactions to the mRNA vaccine, Troelnikov et al. evaluated the ability of PEG, polysorbate 80, BNT162b2 vaccine, and AZD1222 vaccine to activate basophils and mast cells in patients with a previous allergic history of PEG. The authors clarified that PEG covalently modified on vaccine LNP carriers was a potential factor that triggered allergic reactions.^178^ For the allergic reaction caused by mRNA vaccines, molecules with better biocompatibility and lower immunogenicity should be considered vaccine carriers to reduce the rate of hypersensitivity reactions.

##### Autoimmune diseases

Vaccination can trigger a series of immune reactions and the production of neutralizing antibodies against antigens. An excessively strong immune response may simultaneously produce antibodies targeting normal organs or tissues, leading to autoimmune diseases like hepatitis and autoimmune thyroid diseases.

Lodato et al.^156^ reported that two days after the second dose of the BNT162b1 vaccine, a 43-year-old woman developed jaundice. A liver biopsy revealed moderate portal inflammatory infiltration, accompanied by bile duct injury and hepatic lobular punctate necrosis. After eight weeks of corticosteroid treatment, the clinical indices of the liver returned to normal. Given the beneficial effect of steroid treatment and the overall period from vaccination to onset consistent with the progress of the immune response, the patient was diagnosed with autoimmune hepatitis. Furthermore, the causal relationship between vaccine injection and autoimmune hepatitis has not yet been fully determined.

In addition to autoimmune hepatitis, cases of immune hypothyroidism caused by vaccination have been reported. Two female medical staff members showed increased thyroid hormone secretion and elevated thyroid antibody levels three days after receiving the COVID-19 vaccine, indicating inhibited thyroid functions.^157^

The relationship between autoimmune diseases and COVID-19 vaccines has not been clarified. However, the above cases emphasize the importance of regular follow-up and close observation of the physical condition of vaccines. While vaccination is an effective weapon in ending the COVID-19 epidemic, immune-related complications need to be considered.

#### Nervous system diseases

##### Facial paralysis

Bell’s palsy, also known as acute peripheral facial paralysis of unknown cause, is usually characterized by sudden unilateral facial paralysis.^159^ This type of nerve paralysis is typically temporary. Most patients recover within 6–9 months without drug or steroid treatment,^179^ but a few patients may have facial dysfunction. Facial paralysis may occur after vaccination, such as the influenza vaccine, caused by viral reinfection.^180^ In a clinical trial of the COVID-19 mRNA-1273 vaccine, three of 15,210 subjects developed facial paralysis.^114,118^ Wan et al. used the reporting systems of medical institutions to evaluate the proportions of facial paralysis within 42 days after vaccination with BNT162b2 and CoronaVac vaccines and found that they were 66.9 cases/100,000 individuals/year in CoronaVac recipients and 42.8 cases/100,000 individuals/year in BNT162b2 recipients, respectively. A higher proportion of facial paralysis occurred in inactivated vaccine recipients,^159^ indicating that this complication may be related to the vaccine adjuvant as the inactivated vaccine is unlikely to cause virus reinfection and does not contain active viral nucleic acid. Renoud et al. conducted a disproportionate data analysis based on the WHO pharmacovigilance database and found that 844 cases among 133,883 mRNA vaccination cases had facial paralysis-related events.^181^ Although the COVID-19 vaccine may cause acute peripheral facial paralysis, the beneficial and protective effects outweigh the risk of this generally self-limiting adverse event. Adverse event monitoring and controlling should be improved and strengthened to ensure a timely treatment in case of complications.

##### Functional neurological disorder (FND)

FND is a nervous system disease that can produce neurological symptoms caused by biological, psychological, or environmental factors.^153^ The predisposing factors for FND include head injury, surgery, and vaccination. Currently, at least one vaccinated individual has been diagnosed with FND. Kim et al.^153^ described the potential relationship between FND and COVID-19 vaccination. Vaccine components are unlikely to be the main cause of FND because FND also occurs after normal saline injection.

Moreover, adverse events, such as local pain at the injection site or systemic muscle pain, may occur after vaccination, which may increase the sensitivity of the patient’s nerves. The reason for FND attacks caused by COVID-19 vaccines has not been determined. Close attention should be paid to the adverse events of vaccinated individuals. Improving the reporting of such events, the public’s confidence in the government and medical institutions will greatly reduce recipients’ psychological and mental pressure, reducing the incidence of FND.

#### Lymphatic diseases

Injection of the COVID-19 vaccine may lead to inhibition of thyroid function. Since the time window from vaccination to the disease is consistent with the immune process, such adverse reactions are classified as immune diseases, namely autoimmune diseases. In addition, lymphatic diseases, such as abnormal lymph nodes,^160^ may also occur after receiving the COVID-19 vaccine. For example, three days after receiving the first dose of the AZD1222 vaccine, eosinophils were detected in the left axillary lymph nodes of a 75-year-old male using [18 F] Choline positron emission tomography/computed tomography (PET/CT), demonstrating the mild uptake ability of choline. The choline uptake occurred in his left arm 3 days after AZD1222 vaccination, indicating the AZD1222 vaccine-induced abnormal lymph node exists. Eifer et al. also described that a 72-year-old woman vaccinated with BNT162b2 subsequently displayed the same phenomenon of increased choline uptake by lymph nodes.^182^ The vaccine recipients had tumors resected or treated by other means in both cases. [18 F] Choline PET/CT is an effective method to determine the location of tumor infiltration and the prognosis of tumor patients. Therefore, close follow-up of patients with tumors inoculated with the COVID-19 vaccine should be prudent to avoid incorrect interpretation of the imaging results and incorrect diagnoses of diseases.

#### Other diseases

In addition to the diseases mentioned above, some COVID-19 vaccines recipients may also have skin diseases, including Rowell syndrome,^161^ macula,^162^ and chilblain-like lesions.^163^

Gambichler T et al.^161^ found that a 74-year-old woman developed a severe rash one day after receiving the BNT162b2 vaccine. Clinical examinations showed that the patient had red cohesive spots and papules on the trunk and limbs but no mucosal infiltration. The patient was diagnosed with Rowell’s syndrome (RS), a relatively rare disease characterized by lupus erythematosus with pleomorphic erythematosus lesions and immunological manifestations through further skin biopsy.^183^ Subsequently, the patient received steroid treatment, and the symptoms were relieved. In this case, the BNT162b2 vaccine was considered a possible cause of RS, but the patient took pantoprazole for a long-time treatment of chronic gastrointestinal ulcers. Combining this drug and the COVID-19 vaccine may lead to the onset of RS. Some studies have pointed out that omeprazole, a proton pump inhibitor, may cause RS.^184^ Therefore, special vaccination groups, especially the elderly or patients with underlying diseases, should be paid attention to their post-vaccination status, and corresponding treatment should be given in time.

Jedlowski P et al.^185^ have reported a measles-like rash and papules caused by the BNT162b2 vaccine. After the first dose of the vaccine, a 30-year-old male had adverse reactions such as fever and pain at the injection site, followed by a measles-like rash. After the second dose of the vaccine, he had a recurrent measles-like rash and flesh-colored papules, which had subsided after corticosteroid treatment. Similarly, a 55-year-old man suffered pain and pruritus erythema at the injection site after the first dose of the BNT162b2 vaccine, accompanied by impaired liver function.^162^ Subsequently, the patient’s symptoms were significantly improved after corticosteroid therapy.

Piccolo et al. noted that a 41-year-old woman had chilblain-like lesions (CLL) on her fingers and was accompanied by severe pain after receiving the second dose of the BNT162b2 vaccine.^163^ This symptom is most likely related to the strong activation of innate immunity and the production of potent antibodies.^186^ Additionally, CLL was observed in another 41-year-old female vaccinee, accompanied by severe pain.^187^ Although the reasons for CLL in the above cases have not been clarified, the occurrence of CLL after the COVID-19 mRNA vaccine proves the correlation of CLL with the vaccination.^186^

In conclusion, although COVID-19 vaccination may be associated with diseases such as thrombosis, myocarditis, and allergy, the proportion of adverse events is low, and vaccination is still an effective means to control and block the epidemic.

### Effect of COVID-19 vaccination in different populations

**Fig. 9:**
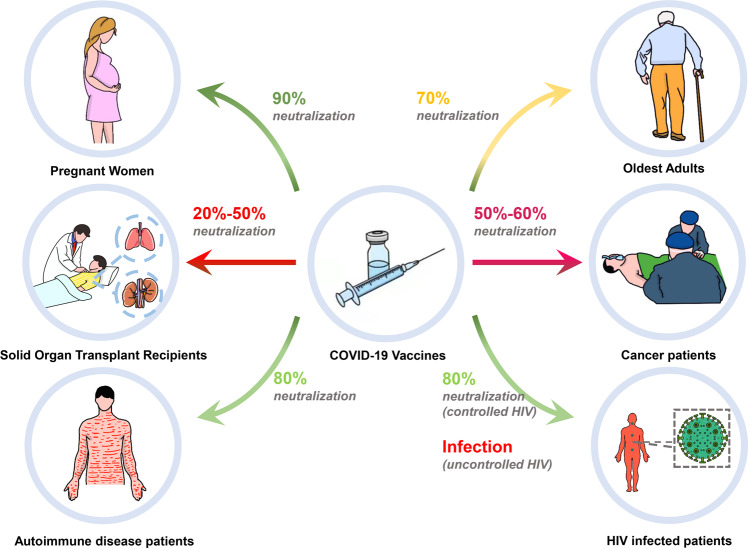
Effect of vaccination in different populations. COVID-19 vaccines are still effective for pregnant women, patients with autoimmune diseases, and controlled HIV-infected patients, and the overall efficacy can maintain about 80–90%, while the 30% neutralization reduction occurs in older people. Moreover, the overall neutralizing activity of COVID-19 vaccines in solid organ transplant recipients, cancer patients, and uncontrolled AIDS patients is significantly reduced

COVID-19 vaccine mainly functions by inducing neutralizing antibodies and memory cells. However, for patients with innate immune diseases, such as autoimmune rheumatism and a history of allergies or tumors, COVID-19 vaccination may cause adverse events. In addition, elderly and pregnant women are also of concern. Compared to adults, vaccine immunization of the elderly may not achieve the desired protective effect due to their weakened immune system functions.^188–190^ For pregnant women, the COVID-19 vaccine may cause adverse events, such as abortion, premature birth, or fetal malformation.^191,192^ Here, we summarize the effects of vaccination in different populations (Fig. 9).

#### Pregnant women

Previous studies have shown that complications including lung injury, diabetes, and cardiovascular diseases in pregnant women after SARS-CoV-2 infection are higher than that in non-pregnant women.^193^ However, adverse events, such as abortion or fetal malformation, may occur after COVID-19 vaccination,^191,192^ which have raised concerns. Shimabukuro et al.^192^ evaluated the effects of COVID-19 vaccination on pregnant women and fetuses using the V-safe monitoring and VERS systems. The results indicated that adverse reactions were higher in pregnant women than in non-pregnant women. The most significant adverse event was pain at the injection site. After mRNA vaccination, pregnancy loss occurred in 13.9% of the pregnant women, 86.1% had a normal pregnancy, and 9.4% had a premature delivery. Although pregnancy loss and premature birth could occur, both are low-probability cases, and the benefits of vaccination far outweigh the risks. In addition, the proportion of local or systemic adverse reactions in elderly non-pregnant women was similar to that in pregnant women,^191^ indicating that physiological changes during pregnancy did not significantly impact the occurrence of adverse events.

Two other studies analyzed the immunogenicity of COVID-19 in pregnant women and fetuses, and COVID-19 vaccines overall are approximately 90% effective for the vaccinated women.^194,195^ R Collier et al. analyzed the immune condition of pregnant or lactating women and fetuses after COVID-19 vaccination.^194^ Both pregnant and lactating women could produce binding, neutralizing, and functional non-neutralizing antibodies, accompanied by CD4^+^ and CD8^+^ T-cell responses. More importantly, binding and neutralizing antibodies were also detected in infant umbilical cord blood and breast milk. These results show that vaccinated pregnant women experience a personal protective effect and produce antibodies that can be delivered to the fetus through the umbilical cord or breast milk to provide immune protection.

Furthermore, a multicenter study conducted in Israel also showed that after vaccination with the BNT162b2 vaccine, IgG antibodies could be produced in the mother. These antibodies can pass through the fetal barrier, and newborns can detect antibody reactions.^195^ These two studies showed that after the COVID-19 vaccination, the antibodies in pregnant women could be transferred into the fetus through efficient mother-to-child transmission, effectively protecting the fetus.

Although pregnant women are more likely to experience adverse events after vaccination than non-pregnant women, this proportion is still limited. Within the ideal range, the COVID-19 vaccine can simultaneously protect mothers and infants, reducing the probability of fetal infection with SARS-CoV-2 after birth to a certain extent. Therefore, pregnant women should be voluntarily vaccinated with the COVID-19 vaccine. Meanwhile, government and medical institutions should further improve the health monitoring of pregnant women in the trial to ensure the safety of pregnant women and fetuses.

#### Elderly individuals

Several studies have analyzed the related immunization levels in the elderly (> 80 years of age) after the COVID-19 vaccination. About 70% protection suggested that at least two vaccination doses should be given to these people.^189,190^ Lisa et al.^190^ compared the production of serum neutralizing antibodies between elderly (>80 years old) and young (<60 years old) vaccine recipients after vaccination with BNT162b2. The IgG antibody titer of the elderly subjects was generally lower than that of the young subjects. Although the antibody levels increased after secondary immunization, 31.3% of the elderly did not produce SARS-CoV-2 neutralizing antibodies, while the antibodies were not detected in only 2.2% of the young subjects after the second dose. Because virus variants, especially variants of concern (VOC), have stronger infectivity or immune escape ability and are prevalent globally. Collier et al.^189^ evaluated the effect of serum neutralizing antibodies in elderly individuals on VOC strains Alpha, Beta, and Gamma after two doses of the BNT162b2 vaccine. Neutralizing antibodies against the VOC strain were detected in all age groups. Therefore, the COVID-19 vaccination can still protect the elderly. However, compared with young vaccinated individuals, the CD4^+^ T-cell response of elderly participants was poor and manifested as low levels of IFN-γ and IL-2. Consequently, government and medical institutions should conduct long-term monitoring of the elderly population and timely deliver “booster shot” vaccination or increase the vaccine dosage to maintain immune efficacy.

Although the COVID-19 vaccine is an effective method to control the pandemic, the current global vaccine resources are still relatively scarce, and complete immunization has not been achieved in most countries. Shrotri et al.^196^ conducted a prospective cohort study to systematically analyze the protective effect of a single dose of AZD1222 or BNT162b2 vaccine in individuals aged ≥ 65. After the first dose of the vaccine, evident protection for the elderly lasted for at least 4 weeks, and SARS-CoV-2 transmission was reduced to a certain extent. Another study showed that a single dose of the COVID-19 vaccine could reduce the risk of hospitalization in elderly patients infected with SARS-CoV-2.^197^

The collective findings support the view that the elderly should be actively vaccinated against COVID-19. If two doses of vaccine cannot be administered, they should be vaccinated with a single dose. The COVID-19 vaccine can reduce the risk of SARS-CoV-2 transmission to a certain extent, decrease the risk of hospitalization, and promote the safety of the elderly.

#### Organ transplant recipients

To reduce the immune system’s recognition and attack, patients with solid organ (e.g., kidney and heart) transplantation require long-term immunosuppressants, such as tacrolimus, corticosteroids, and mycophenolate organs.^198^ Although immunosuppressive drugs can maintain transplanted organs, they may also affect the body’s antiviral immunity, making solid organ transplant patients more susceptible to SARS-CoV-2 infection and increased mortality risk.^198^

Effective immunization of this population is necessary to reduce the infection and death caused by SARS-CoV-2. Several studies have reported that the efficiency of COVID-19 vaccines in solid organ transplant patients after single-dose/two-dose vaccination and enhanced immunization (third dose) was only 20–50%.^199–201^ Boyarsky et al. evaluated the effect of a single dose of BNT162b2 or mRNA-1273 vaccine in organ transplant patients.^199^ Only 76 (17%) of the 436 subjects elicited neutralizing antibodies, and the titer of these antibodies in elderly patients was lower than that in young individuals. Individuals vaccinated with mRNA-1273 produced higher levels of antibodies. These results showed that a single dose of the COVID-19 vaccine could not effectively prevent SARS-CoV-2 infection in organ transplant patients. Subsequently, this group analyzed two vaccine doses in 658 organ transplant patients.^200^ 15% of the subjects produced neutralizing antibodies after the first dose of vaccine, whereas 54% after the second dose, indicating that complete vaccination should be fully deployed for organ transplant patients and that these individuals should be closely monitored after vaccination to prevent SARS-CoV-2 infection. Another study carried out by Benotmane I et al. showed that after the third dose of the mRNA-1273 vaccine, neutralizing antibodies were detected in the serum of 49% of renal transplant patients.^201^ However, some patients still did not produce neutralizing antibodies, especially those receiving triple immunosuppressive therapy with tacrolimus, corticosteroids, and mycophenolate mofetil after vaccination. In addition to the mRNA vaccine, the protective effect of an inactivated vaccine—the CoronaVac vaccine on organ transplant patients was also evaluated 31 days after two doses.^198^ Sixteen of the 85 renal transplant patients had neutralizing antibody reactions.

Furthermore, this result may be related to some participants’ small sample size and impaired renal function. Monitoring neutralizing antibody levels in organ transplant patients should be strengthened, and a booster shot should be administered in time. Mazzola et al.^202^ assessed antibody levels in other organ transplant patients after two doses of the BNT162b2 vaccine. In liver, kidney, and heart transplant patients, serum conversion rates were 37.5, 16.6, and 34.8%, respectively. The lower neutralization level in kidney transplant patients was consistent with the study by Sadioğlu et al.^198^

The collective findings support the view that for solid organ transplant patients who take immunosuppressants, timely vaccination is important, and clinicians should closely monitor their appropriate antibody levels. Based on the actual situation of this population, immunosuppressive programs and vaccination countermeasures should be formulated to reduce SARS-CoV-2 infection rates.

#### Cancer patients

Besides organ transplant patients, cancer patients are also a COVID-19 high-susceptible population. Anti-tumor treatments, including radiotherapy and chemotherapy, may lead to systemic hypoimmunity.^203^ Several studies have indicated that vaccination can protect about 50–60% of cancer patients from the SARS-CoV-2 infection; thus, they should receive COVID-19 vaccines as soon as possible and complete at least two doses of injection.^204–206^

Monin et al.^204^ evaluated the safety and immunogenicity of a single dose and two doses of the BNT162b2 vaccine in cancer patients. Twenty-one days after the first dose of the vaccine, 21 of the 56 patients with solid tumors and eight of the 44 patients with blood cancer displayed an anti-S protein immune response. These findings showed that a single dose of the COVID-19 vaccine could not effectively prevent cancer patients, especially those with blood cancer, from the infection with SARS-CoV-2. In contrast, 18 patients with solid cancer and three patients with blood cancer were seroconverted after the second dose of the vaccine. In addition, the BNT162b2 vaccine was safe for patients with breast and lung cancer, and no death caused by vaccination was reported during the trial.

Similarly, Palich et al. evaluated the neutralization activity of the BNT162b2 vaccine in patients with cancer.^206^ The seroconversion rate after vaccination was only 55%. Terpos et al.^207^ and Maneikis et al.^208^ studied the effectiveness of the BNT162b2 vaccine in elderly patients with multiple myeloma and hematological malignancies, respectively. After the first dose of the vaccine, low levels of neutralizing antibodies were detected in the serum of the myeloma patients, which may be due to the inhibition of B-cell proliferation and antibody production by myeloma cells. Patients with hematological malignancies who received two doses of the BNT162b2 vaccine could display serious SARS-CoV-2 breakthrough infections since malignant hematological tumors can destroy immune homeostasis, and the immunosuppressive drug used in the treatment can also affect the production of neutralizing antibodies.

The above studies demonstrate that patients with malignant tumors are susceptible to COVID-19 and should receive timely vaccinations. The vaccination schedule should be based on the patient’s antibody titers to appropriately shorten the interval between the two vaccine injections^205^ and ensure a strong immune response. Moreover, patients with malignant tumors should be closely monitored after receiving the COVID-19 vaccine to prevent serious breakthrough infections.

#### Human immunodeficiency virus (HIV) infected persons and patients with autoimmune diseases

Organ transplant patients and tumor patients may be affected by immunosuppressive drugs and systemic hypoimmunity.^198,207^ In addition, HIV-infected and autoimmune disease patients are also susceptible to SARS-CoV-2 infection due to their impaired immune system function and immunosuppressants. Several studies have shown that the overall efficacy of the COVID-19 vaccine in controlled HIV-infected people and people with autoimmune disease was about 80%, while the vaccination could not prevent the breakthrough infection in patients with progressive AIDS.^209,210^

In one study, the AZD1222 vaccine induced strong neutralization reactions in HIV-negative individuals and AIDS patients with well-controlled infections after receiving antiretroviral therapy (ART).^27^ Fourteen days after the second dose of the AZD1222 vaccine, HIV-negative individuals and HIV-positive patients treated with ART showed similar neutralizing antibody levels, and antibodies were detected in 87% (13/15) of HIV-infected persons. The results indicate that for HIV patients receiving ART, COVID-19 vaccination can produce an immune response similar to HIV-negative individuals. In contrast, for HIV patients whose condition is not effectively controlled, especially those with progressive AIDS, two doses of the vaccine may not prevent breakthrough infection.^209^

In addition to individuals infected with HIV, patients with autoimmune diseases (e.g., autoimmune rheumatism) may also get impaired immunity from the COVID-19 vaccine because of their medication with immunosuppressants, such as mycophenolate mofetil and corticosteroids.^210^ In one study, after two doses of the BNT162b2 vaccine, 86% of patients with autoimmune rheumatism experienced serum transformation, but the levels of S1/S2 neutralizing antibodies were significantly lower than that in healthy individuals. Some patients with enteritis who received immunosuppressive treatment also showed reduced immunogenicity following the BNT162b2 and AZD1222 vaccines.^211^ These findings highlight that immunization should be completed promptly for individuals receiving the immune drug and that the drug dosage should be adjusted appropriately during vaccine injection to ensure the production of neutralizing antibodies.

### Antibody-dependent enhancement (ADE) of vaccines

ADE is a phenomenon in which the pathogenic effect of some viral infections is strengthened in sub-neutralizing antibodies or non-neutralizing antibodies.^212–214^ In other words, after natural immunization or vaccination, when contacting the relevant virus again, the antibody produced before might enhance the infection ability of the virus and eventually aggravate the disease. Currently, there is no definitive mechanism to explain the causes of this phenomenon.^215^ The ADE simulated in vitro attributes to the pathogenic mechanism as follows: (1) The entry of virus-mediated by the Fcγ receptor (Fcγ R) increases viral infection as well as replication;^216,217^ (2) Excessive antibody Fc-mediated effector functions or immunocomplex formation enhances inflammation and immunopathology.^214,215^

Previous studies have shown that HIV, Ebola, influenza, and flaviviruses may induce ADE.^215^ And it was reported that respiratory syncytial virus and dengue virus vaccines could also cause ADE, so it is necessary to evaluate the ADE risk of COVID-19 vaccines.^218^ Although no serious ADE event caused by the COVID-19 vaccine has been released,^217^ the data obtained from other coronaviruses like SARS-CoV and MERS-CoV vaccines can provide experience.^215^

Pathogen-specific antibodies that can promote the incidence of pathological ADE should be considered during the development of COVID-19 vaccines. In vitro studies of antibodies against viral infection have identified factors associated with ADE, such as insufficient concentration or low-affinity antibodies.^18^ However, protective antibodies may also induce ADE. For instance, the antibody against feline infectious peritonitis virus also enhances infection of monocytes,^214^ and data from SARS-CoV or other respiratory virus studies suggest that SARS-CoV-2 antibodies may exacerbate COVID-19.^217^ Clinical studies have shown that SARS-CoV-2 antibodies can bind to mast cells, which may be related to the multisystem inflammatory syndrome in children (MIS-C) and multisystem inflammatory syndrome in adults (MIS-A) after COVID-19.^219^ The binding of SARS-CoV-2 antibodies to Fc receptors on macrophages and mast cells may represent two different mechanisms of ADE in patients. The above findings indicate the possibility of ADE induced by COVID-19 vaccines, to which more attention should be paid to.^220^

The preclinical results suggest that vaccination with formalin-inactivated SARS-CoV virions, MVA vaccine expressing SARS-CoV S protein, and S-derived peptide-based vaccine may induce lung disorders in the NHP model.^214^ When macaques were inoculated with inactivated SARS-CoV vaccine, they showed ADE after viral infection, manifesting as extensive macrophage and lymphocyte infiltration in the lungs and edema in the alveolar cavity. Mice and hamsters inoculated with trimeric S protein vaccine were not infected with SARS-CoV, but the serum produced could promote the entry of ACE2-independent pseudovirus.^221^ Rhesus monkeys inoculated with a high dose of COVID-19 vaccine had elevated body temperature within 1 day, increased respiratory rate, and decreased appetite within 9–16 days.^216^ Monkeys euthanized on days 3 and 21 displayed multifocal lung injury, alveolar septum thickening due to edema and fibrin, the slight appearance of type II lung cells, and perivascular lymphocyte proliferation.^214^

These models and data emphasize the importance of developing a safe anti-antibody-independent COVID-19 vaccine. At the same time, it is necessary to pay close attention to ADE caused by vaccination against COVID-19. Some studies have shown that antibodies with low affinity and poor neutralization ability may aggravate this disease, while current clinical markers cannot distinguish between severe infection and enhanced antibody dependence.^214,218^ Therefore, data and mitigation methods from SARS-CoV and MERS-CoV are referential to analyze the ADE phenomenon caused by COVID-19 vaccination. It is important to develop better COVID-19 vaccines and immunotherapy, overcome the identified mutants, and reduce possible ADE pathology.

## Improvement of COVID-19 vaccines

Although COVID-19 vaccines can reduce the risk of infection and the mortality of patients, problems with the vaccines at present include declining neutralization activity of variants and vaccination-related adverse events.^14,153,222^ Adopting mix-and-match vaccines^223^ and developing new vaccines, such as VLPs and nanoparticle vaccines,^224^ improving existing vaccine adjuvants,^225^ and changing the vaccination route^226^ might enhance the efficacy of vaccines and reduce the occurrence of adverse events to some degree (Fig. 1).

### Mixed inoculation

In the absence of available vaccine resources, the second injection of an allogeneic vaccine may effectively advance the immunization process. However, vaccination with non-homologous vaccines may raise concerns about safety and effectiveness. Borobia et al. assessed the immunogenicity after inoculating a heterogeneous COVID-19 vaccine and indicated that the heterogeneous vaccine might provide greater immune protection. An initial dose of AZD1222, followed by the BNT162b2 vaccine, can induce strong immune responses and is safe.^227^ The research of Hillus et al.^228^ reached a similar conclusion. Compared with two doses of AZD1222 administered 10–12 weeks apart and BNT162b2 administered 2–3 weeks apart, the AZD1222 and BNT162b2 vaccines administered at an interval of 10–12 weeks were more effective, with better tolerance and immunogenicity. Heterologous vaccination can complement the advantages of different vaccines,^229^ as vaccination with BNT162b2 can elicit strong B-cell immunity and induce high levels of neutralizing antibodies, whereas the AZD1222 vaccine can induce strong T-cell responses. Therefore, this scheme is suitable for individuals with decreased immune function (e.g., organ transplants and cancer patients). Several studies evaluated the neutralization activity of the Omicron variant by the booster dose of homologous or heterologous inoculation.^230,231^ Both homologous and heterologous enhancers could increase the neutralization activity of subjects’ serum against the Omicron variant, but the neutralization efficiency of an additional heterologous vaccine was higher, supporting the sequential vaccination with heterologous vaccines.

In addition, several studies have shown that individuals previously infected with SARS-CoV-2 have a stronger immune response after the vaccination.^138,232–234^ Planas et al. tested the serum and antibody levels of 21 medical staff infected with SARS-CoV-2 12 months before vaccinating with a single dose of COVID-19 vaccine (vaccinated 7–81 days before sampling).^138^ The serum effectively neutralized Alpha, Beta, and Delta variants, and similar results were obtained by Mazzoni et al.^232^ After a single dose of the vaccine, the cellular and humoral immunity levels of patients who had rehabilitated from COVID-19 were further strengthened,^233^ and memory B-cell responses were significantly enhanced. These findings explain the significant increase in antibody levels after the first vaccination of rehabilitation patients.^24^ Havervall et al. showed that a single dose of COVID-19 vaccine could be used as an effective immune enhancer within at least 11 months after being infected with SARS-CoV-2.^234^ Liu and colleagues evaluated the efficiency of the BNT162b2 booster dose against B.1.1.529 (Omicron) variant and found that the serum neutralizing antibody levels from previous-infected recipients with booster dose is higher than naive-uninfected counterparts.^235^

The collective findings support the view that vaccination should be actively carried out, regardless of whether the individuals have been infected with SARS-CoV-2 or not. Although previously infected individuals are better protected after a single dose of vaccine, the possibility of breakthrough infection still exists as this immune enhancement may be related to the body’s level of memory B cells.^24^ However, there may be individual differences in the level of memory B cells. Therefore, regular antibody testing should be performed for rehabilitated persons who have received a single dose of vaccine to ensure lasting immunity. In addition, it is also a feasible method to implement heterologous vaccination in case of a vaccine shortage. The mixed-vaccination results of CoronaVac and ZF2001 vaccines also supported this view, as the former is much safer while the latter has better immunogenicity.^236^ In addition, Zhu et al. found that the mix-vaccination of CoronaVac and Ad5-nCoV can induce higher neutralizing antibodies and provide more effective protection than homologous vaccination.^237^

### Nanoparticle vaccines

New vaccine platforms, such as mRNA vaccines, provide more powerful immune protection than traditional vaccines. However, these vaccines have lower neutralizing activity against variants, especially the Beta and Delta.^14,222^ Nanoparticle vaccines may have better neutralizing activity than mRNA vaccines,^224,238,239^ providing a new direction for vaccine development.

Ko et al.^224^ designed a nanoparticle vaccine consisting of 24 polymer SARS-CoV-2 RBD nanoparticles and a ferritin skeleton. The vaccine caused cross-neutralizing antibody reactions to bat coronavirus, SARS-CoV, and SARS-CoV-2, including Alpha, Beta, and Gamma variants. The DH1041-DH1045 potent neutralizing antibody induced by the vaccine had neutralizing activity against various mutations, including K417N, E484K, and N501Y. Walls et al. designed a self-assembled protein nanoparticle immunogen composed of 60 SARS-CoV-2 S protein RBDs. The immunogen can target different immune epitopes and still induce high levels of neutralizing antibody expression at low doses.^239^ Moreover, compared with traditional vaccines, nanoparticles can exist in B-cell follicles for a long time, producing a sustained germinal center reaction to ensure the high-level production of antibodies.^238^ In addition, according to the self-assembly function of ferritin, S protein RBD,^224^ hemagglutinin,^240^, and other important viral proteins can be inserted and act as the physiologically relevant trimeric viral spike form to further improve the vaccine efficacy.^238^ Therefore, by optimizing the packaging of antigens and producing a stronger, longer-lasting immune response, nanoparticle vaccines are likely to play an important role in future COVID-19 vaccines.

### Improvement of immune adjuvants

An adjuvant is a vaccine component to enhance the immune response, playing a very important role in improving the efficacy of vaccines and reducing adverse events to ensure safety.^225,241^ In the past two decades, a series of new adjuvants have been used in licensed vaccines, including Aluminum hydroxide, MF59, AS03, CpG 1018, and CoVaccine HT,^241^ among which the Aluminum hydroxide can reduce the immune-related pathological reactions while other adjuvants can trigger specific cell receptors and induce an innate immune response in the injection site as well as the draining lymph nodes, further promoting the production of antibodies.^225,242^ Therefore, appropriate adjuvants are critical for maintaining vaccines’ durability and effectiveness. Here, some brief information on existing adjuvants used in COVID-19 vaccines is provided in Table 4.

**Table 4:** Adjuvants used in COVID-19 vaccines in the clinic

Adjuvant	Components	Receptor/pathway	Vaccine	Ref
Alum	Aluminum salts	NLRP3 uric acid, DNA	BBIBP-CorV, CoronaVac (Inactivated SARS-CoV-2 virus vaccines)	^38,225^
Matrix-M/ IscoMatrix	Saponin	Unknown	NVX-CoV2373 (Recombinant SARS-CoV-2 S protein)	^31^
MF59	Squalene oil, surfactant	IL-4, STAT6	Recombinant SARS-CoV-2 S protein as a soluble protein or on virus-like particles	^245^
AS03	Squalene oil, Surfactant, α- tocopherol	G-CSF	Recombinant SARS-CoV-2 spike S protein as a soluble protein or on virus-like particles	^225^
CpG 1018	Synthetic DNA alone or formulated with Alum	TLR9	Recombinant SARS-CoV-2 S protein on virus-like particles	^225,242,248^
Ligand adsorbed in alum	Ligand adsorbed in alum	TLR7/TLR8	Inactivated SARS-CoV-2 vaccines	^225^

Alum is the most widely used adjuvant in global vaccine development, which can induce the antibody response and different CD4^+^ cell responses (low level).^225,241^ Relevant mechanisms can be explained as enhancing anti-phagocytosis and activating the proinflammatory NLRP3 pathway.^242^ In addition, Aluminum adjuvants can reduce immune-related pathological reactions and improve safety, explaining the excellent safety of BBIBP-CorV and CoronaVac (both of the vaccines used Aluminum hydroxide as adjuvants).^221,243^ However, the immunogenicity of aluminum adjuvant is poor. The chemical modification of alum with short peptide antigens composed of repeated serine phosphate residues can significantly enhance GC cell and antibody responses.^244^

MF59 is a squalene oil-in-water emulsion adjuvant approved for use in influenza vaccines in more than 38 countries, and it is biodegradable and biocompatible.^245^ MF59 showed good tolerance and safety, and the inoculation of vaccines that use this adjuvant can motivate the activation of macrophages and the production of chemokines. These chemokines will recruit neutrophils, eosinophils, and monocytes to the lymph nodes, further form a cascade amplification reaction, and activate B cells and T cells.^225^ In addition, MF59 can stimulate IL-4 and STAT6 signal pathways and induce the antibody response. It is worth noting that the above response does not depend on type 1 interferon or inflammatory pathway.^246^ Thereby, MF59 has been selected as the adjuvant of COVID-19 vaccines.

AS03 is similar to MF59 but has an additional immune-enhanced component α- tocopherol (vitamin E). Thus, it can induce the expression of proinflammatory cytokines and chemokines independently (not depending on the type I interferon).^242^ In addition, AS03 can trigger a transient innate immune response, the injection of AS03 induces the transient production of cytokines in the mice model, and vitamin E can further enhance the expression of some chemokines and cytokines like CCL2, CCL3, and IL-6.^225^ AS03 is evaluated as the adjuvant of several recombinant S protein vaccines in the clinical trial, the add of AS03 further improve Th2-unbiased cell responses and the production of IFN-γ, which may enhance the efficacy of COVID-19 vaccines.^247^

CoVaccine HT is also an oil-in-water (O/W) emulsion, while CpG is a synthetic DNA sequence containing an unmethylated CpG sequence.^242,248^ Compared with the aluminum hydroxide adjuvant, AMP-CpG and CoVaccine HT showed better immunogenicity.^249^ Using AMP-CpG as an adjuvant, persistent antibody and T-cell reactions were still induced in elderly mice at low-dose S protein levels. Reducing the dose of S protein may decrease the occurrence of adverse events and improve vaccine safety. Compared to aluminum hydroxide, CoVaccine HT can promote the production and maturation of neutralizing antibodies to a greater extent, thereby quickly inducing an immune response to SARS-CoV-2.^248^

The use of aluminum adjuvants may reduce the adverse events of related vaccines and improve vaccine safety. However, the immunogenicity of aluminum adjuvants is poor. Therefore, the common use of different adjuvants may improve immunogenicity while ensuring subjects’ safety.

### Change of inoculation route

In addition to sequential immunization (mixed-vaccination), development of new vaccines (such as nanoparticle vaccine), and adjuvant improvement, changing the vaccination route is also a feasible measure to improve the protection and efficacy of existing COVID-19 vaccines.^3,250^ All WHO-approved vaccines adopt the intramuscular route (i.m route), and most of them can only protect the lower respiratory tract except for Ad26.COV-2.S, which can both protect the upper and lower respiratory tract.^48^ However, the new VOC Omicron has stronger infectivity of the upper respiratory tract and mainly causes symptoms of the upper respiratory tract, so the existing vaccine is difficult to protect effectively.^122,235,251^ Mucosal immunity plays an important role in preventing pathogen invasion. The intranasal administration(inhalation route, i.n route) of vaccines may achieve a better protection effect on preventing SARS-CoV-2 infection (especially Omicron variant).^3,250,252,253^ Compared with the traditional i.m route, the i.n route can effectively induce a local immune response. Vaccine antigen enters the respiratory tract and passes through the mucus layer through inhalation to induce the production of local IgA and provide protection at the pathogen’s entry site.^253^ In addition, the i.n route can induce the production of higher levels of mucosal antibodies. Although some IgG can be detected on the mucosal surface after the intramuscular injection, the lack of mucosal IgA still makes the respiratory tract vulnerable to infection.^3^ In addition, the i.n route has better compliance than the i.m route, and the administration is more convenient. However, the i.n route still has some disadvantages: the systemic immune response induced by this administration method is often lower than that of the i.m route because the titer of the virus may decrease when it is made into aerosol; the i.n route may cause antigen or vaccine adjuvant to enter the central nervous system and cause an adverse reaction; and i.n route usually needs auxiliary drug delivery devices (such as pressure device, atomizer), and the cost is higher, which limits the application of this approach.

Among the currently approved inactivated vaccine, viral vector vaccine, protein subunit vaccine, and mRNA vaccine, only viral vector vaccine has the potential to apply intranasal administration because inactivated vaccine, protein subunit vaccine, and mRNA vaccine antigens cannot actively enter cells, so it is difficult to stimulate mucosa effectively, and they remain difficult to commercialize.^250^ Van Doremalen N and colleagues evaluated the efficacy of AZD1222 in macaques and hamsters via intranasal administration. They found that the viral load in the nasal cavity of the experimental group decreased significantly after enhanced intranasal inoculation. No virus particle or RNA was detected in the lung tissue, indicating that intranasal administration is a prospect route for COVID-19 vaccines.^254^ Wu S et al. evaluated the safety, tolerability, and immunogenicity of the aerosolized Ad5-nCoV. The inhalation group(2 doses via i.n route on days 0 and 28) reported fewer adverse events compared with the injection group(2 doses of Ad5-nCOV via i.m route on days 0 and 28) and the mixed group(1 dose via i.m route on day 0 and the second dose via i.n route on day 28). The mixed group showed the highest induced-immune level, but the antibodies produced by the inhalation group were less than those of the injection group, suggesting that the inhalation route of Ad5-nCoV is an effective measure to boost immunity.^226^

The above study shows that the i.n route can protect the upper respiratory tract and inhibit virus infection more effectively than the i.m route, and relevant adverse events are fewer. However, the immune response induced by the i.n route alone is lower than that induced by the i.m route. Thus i.n route is more suitable for strengthening immunity. Through the mix route (i.m route at first and then i.n route), higher levels of antibodies can be induced compared with the repeat i.m route and provide stronger protection. As more and more vaccines are approved for clinical trials, the i.n route will be used more widely.

## Prospects and perspectives

More than 153 candidate vaccines have entered human clinical trials. New vaccine platforms will undoubtedly be evaluated, such as nanoparticle and VLP vaccines.

After vaccination against COVID-19, T-cell immunity (such as the Th1 cell response), B-cell immunity (such as the germinal center response), and other immune responses may be produced.^19,21^ Differentiated Th cells can enhance the immune response in the body by promoting the activation of CD8^+^ T cells and secreting IFN-γ.^31^ With the aid of Th cells, activated B cells proliferate and divide in lymphatic follicles to form germinal centers, eventually form plasma cells, and memory B cells secret high-affinity antibodies. In addition, COVID-19 vaccines can produce memory B cells and memory T cells.^24^ The antiviral immune barrier in the host body can be constructed through the combined action of the humoral immune response, cellular immune response, and memory cells.

Although the COVID-19 vaccines have achieved exciting results in both animal studies and clinical trials,^3^ and seven vaccines have been authorized for emergency use by the WHO, adverse events that include pain at the injection site and fever,^114,118^ as well as complications such as coagulation dysfunction,^154^ myocarditis,^74^ immune diseases,^155^ nervous system diseases^159^ and lymphatic system diseases^160^ caused by vaccination, have raised concerns about vaccine safety. Given the low proportion of overall incidence of adverse events of vaccines and the fact that some complications occur mainly in patients with underlying diseases (e.g., cardiovascular diseases and tumors). Governments and relevant agencies are recommended to accelerate the vaccine immunization process. Simultaneously, special attention should be paid to the health status of the recipients, timely treatment of complications, vaccine development, and ensuring the lives and health of patients. In addition, considering the characteristics of some individuals (e.g., the elderly, pregnant women, organ transplant patients, cancer patients, and patients infected with HIV), relevant agencies should closely monitor adverse events and detect antibody titers after immunization.^190^ For organ transplant and cancer patients, the COVID-19 vaccine showed approximately 50% overall protective efficacy due to the continuous use of immunosuppressive drugs, which is unsatisfactory.^201,206^ Those populations are susceptible to SARS-CoV-2 infection, and timely immunization-enhanced measures should be performed to reduce breakthrough infections. For HIV-infected individuals, the viral level in the body should be effectively controlled during vaccination. Otherwise, breakthrough infections may still occur.^27,209^

New SARS-CoV-2 variants like Omicron often have high infectivity and high immune escape ability in the post epidemic era. The existing vaccine strategies are difficult to effectively prevent infections caused by the Omicron variant, which is not only due to the accumulation of more mutation sites in the S protein, but also because the Omicron variant mainly causes upper respiratory tract infection, while the protective antibodies induced by i.m route are often directed at the lower respiratory tract (lung). In this case, changing or adjusting vaccination strategies is very significant to control the infections and alleviate public health pressure. We believe that the following points deserve attention: (1) Although a booster dose can enhance the response of memory cells and increase the antibody titers to produce a stronger protective effect, the fourth dose injection might not effectively Omicron variant infection.^150^ (2) The optimization of COVID-19 vaccines, such as changing the administration route (use the inhaled vaccine and induced mucosal immunity to protect the upper respiratory tract further), developing new vaccines (for inactivated vaccines, the combined use of seed strain of VOCs like Beta + Delta may induce antibodies with multi-epitopes, as well as the use of VOC sequence for mRNA or viral vector vaccines,^151^) and adopting sequential immunization (the use of vaccines developed in different routes like inactivated + viral vector vaccine/mRNA vaccine) will provide better protection than existing vaccination strategies. (3) Although the adoption of inhalable and sequential immunization can improve the efficacy of COVID-19 vaccines, the incidence of adverse reactions of additional Ad5-nCoV was higher than the additional inoculation with homologous inactivated vaccine.^226^ In addition, the inoculation with viral vector vaccines or mRNA vaccines may lead to the complications mentioned above (such as myocarditis and thrombosis). The vaccine’s safety and effectiveness should be balanced. Although the new vaccine platform (such as the mRNA vaccine) may provide more effective protection, its safety is lower than the inactivated vaccine. Suppose multivalent inactivated vaccines like Beta + Delta inactivated vaccine strategies are adopted. In that case, the development can only be carried out after the emergence of a new variant, and the developing speed is lower than the mRNA vaccine uses new variants’ sequences. (4) The emergence of the Omicron variant may indicate the change of the main infection site of SARS-CoV-2 (other VOC usually cause the lung infection except for Omicron), and the symptoms of Omicron infected people are lighter, the hospitalization rate is lower than Delta, infected patients.^255^ In this case, there are many asymptomatic Omicron infected people. Convenient and effective COVID-19 antiviral drugs (especially oral-taken drugs) will greatly alleviate the severe epidemic situation and contribute to the early end of the COVID-19 pandemic.^256^ In addition, Omicron might not be the last VOC, a new recombinant variant Delta 21 J/AY.4-Omicron 21 K/BA.1, also called “Deltamicron”, has appeared in many countries like France and America, and the NTD of Delta combined with the RBD of Omicron may lead to optimization of viral binding to host cell membranes.^257^ Although the detected sequence of Deltacron was lower than Omicron, and the main symptom is mild upper respiratory tract infection, surveillance should be enhanced for this emerging variant.

Furthermore, previously SARS-CoV-2-infected individuals produced high-level antibody responses after a single dose of the COVID-19 vaccine, which may be associated with the strong memory cell response.^24^ For those who have not been infected with SARS-CoV-2, nanoparticle vaccines may be a better choice to bestow immunity to infections by mutant strains. Compared with traditional vaccines, nanoparticles can remain in germinal center B cells and ensure the production of high-level antibodies by generating a sustained germinal center reaction.^238^ In addition to developing new vaccines, adjuvants with better immunogenicity or combined adjuvants may reduce adverse events and improve the vaccine’s protective efficacy.^248^

With the launch of new vaccines and the approval of oral antiviral drugs, such as molnupiravir, the stalemate between humans and SARS-CoV-2 will be broken.^256,258^ A study conducted by Swadling et al. of 58 medical staff with high exposure risk but had not been infected with SARS-CoV-2 found a higher anti-replication transcription complex (RTC) T-cell reaction.^258^ These findings may provide new ideas for vaccine design by targeting RTC and inducing similar T-cell responses. And a nasal-delivery IgY antibody based on SARS-CoV-2 RBD showed multi-protection against Beta, Delta, and Omicron variants in the animal model, which promised to be an additional measure of pre-exposure prophylaxis of SARS-CoV-2 infection.^259^ These new achievements in the pharmaceutical field will undoubtedly become powerful weapons against COVID-19 and help end the pandemic.

## Supplementary information

Language Editing Certificate
