# Supplementary material for: COVID-19 vaccine development: milestones, lessons and prospects
Source: Signal Transduct Target Ther. 2022 May 3;7:146. doi: 10.1038/s41392-022-00996-y (PMC9062866; doi:10.1038/s41392-022-00996-y)
Supplement: Supplementary file 1 — Language Editing Certificate [file 41392_2022_996_MOESM1_ESM.pdf]

ELSEVIER

Certificate of Elsevier Language Editing Services

The following article was edited by Elsevier Language Editing Services:

"COVID-19 vaccine development: milestones, lessons, and prospects"

Authored by:

Huahao Fan

Date: 29-Mar-2022

Serial number: LE-228546-D99F53FBF9AD
